# Fn-Dps, a novel virulence factor of *Fusobacterium nucleatum*, disrupts erythrocytes and promotes metastasis in colorectal cancer

**DOI:** 10.1371/journal.ppat.1011096

**Published:** 2023-01-24

**Authors:** Yixian Wu, Songhe Guo, Fangfang Chen, Yiqiu Li, Yuying Huang, Wanli Liu, Ge Zhang

**Affiliations:** 1 Department of Microbial and Biochemical Pharmacy, School of Pharmaceutical Sciences, Sun Yat-sen University, Guangzhou, China; 2 State Key Laboratory of Oncology in South China, Collaborative Innovation Center for Cancer Medicine, Sun Yat-sen University Cancer Center, Guangzhou, China; Fred Hutchinson Cancer Research Center Arnold Library: Fred Hutchinson Cancer Research Center, UNITED STATES

## Abstract

*Fusobacterium nucleatum* (Fn) is a critical colorectal cancer (CRC)-associated bacterium. DNA hunger/stationary phase protective proteins (Dps) are bacterial ferritins that protect DNA from oxidative stress. However, little is known about the regulatory roles of Fn-Dps towards host cellular functions. Here, we identified Fn-Dps from the culture supernatant of Fn by mass spectrometry, and prepared the recombinant of Fn-Dps protein. We show a novel virulence protein of Fn, Fn-Dps, which lyses and disrupts erythrocytes by the competition for iron acquisition. Also, Fn-Dps facilitates intracellular survival of Fn in macrophages by upregulating the expression of the chemokine CCL2/CCL7. In addition, Fn-Dps can elicit a strong humoral immune response, and mucosal immunization with Fn-Dps conferred protection against Fn in the intestinal tract. Moreover, a high level of anti-Fn-Dps antibody was prevalent in populations, and elevated anti-Fn-Dps antibody levels were observed in CRC patients. Furthermore, Fn-Dps promotes the migration of CRC cells *via* the CCL2/CCL7-induced epithelial-mesenchymal transition (EMT) and promotes CRC metastasis *in vivo*.

## Author summary

Our previous studies have found that the CRC-associated bacterium Fn is a facultative intracellular bacterium. To date, the underlying mechanism by which Fn infection induces carcinogenesis remains largely unknown. Here, our findings revealed that Fn-Dps is a novel multifunctional Fn virulence factor that lyses and disrupts erythrocytes, enhances intracellular survival of Fn in macrophages, and promotes CRC metastasis. We also found that mucosal immunization with Fn-Dps conferred protection against Fn. A high level of serum anti-Fn-Dps antibody was prevalent in populations, and elevated anti-Fn-Dps antibody levels were observed in CRC patients. These findings reveal a novel Fn virulence factor that promotes tumor progression, providing a potential novel drug target for CRC treatment.

## Introduction

*Fusobacterium nucleatum* (Fn) is an opportunistic pathogenic bacterium causing anaerobic infections, such as periodontitis, tonsillitis, appendicitis, and liver abscess.[1,2] Fn was also the most common species identified in *Fusobacterium* bacteremia, [3] in which Fn is mainly associated with comorbidities such as cancer.[4]

In a recent study, Fn has been identified as an important intratumoral bacterium and implicated in the development of various gastrointestinal tumors, including esophageal, gastric, and colon tumors.[1,5,6] Overwhelming evidence has demonstrated that Fn is the most critical microbial risk factor in colorectal cancer (CRC) initiation and progression.[1,7,8]

The virulence mechanisms of Fn responsible for infection and tumors include colonization, dissemination, and induction of host responses.[9] Fn encodes several proteins, including FadA, Fap2, and RadD, for adhesion or invasion of host cells.[10] Moreover, Fn regulates oncogenic responses by microRNAs and immune modulation.[11–13] Recently, Fn was reported to promote CRC metastasis by modulating the ALPK1/NF-κB/ICAM1 axis and activating autophagy signaling.[7,14] We recently confirmed that exosomes released from Fn-infected cells promote tumor metastasis.[15] However, the role and mechanisms of Fn in colorectal carcinogenesis have not been thoroughly elucidated to date.

Previously, our study indicated that Fn is a facultative intracellular bacterium that can survive in macrophages.[16] Although anaerobic, Fn exhibits a high degree of aerotolerance, which enables it to survive in periodontal pockets that are occasionally exposed to aerobic conditions.[17] Strikingly, obligate anaerobes have manifested H_2_O_2_-inducible defenses using mechanisms nearly identical to aerobic organisms.[18] DNA hunger/stationary phase protective proteins (Dps) are resistant to multiple environmental stresses and can attenuate the production of reactive oxygen species (ROS). ROS are essential components of the innate immune response against intracellular bacteria.[19] Although numerous studies have identified the role of Dps in protecting bacteria during infection, little is known regarding the role of Dps in host cell responses.

In the present study, we investigated the Dps homolog Fn1079 (Fn-Dps), a novel virulence factor of Fn, and examined gene expression changes in Fn-Dps treated cells. Moreover, we evaluated the efficacy of Fn-Dps as a target protein for diagnostic and therapeutic applications, and described the role of Fn-Dps in CRC progression.

## Materials and methods

### Ethics statement

Human serum samples from healthy subjects (HS, n = 144) and colorectal cancer patients (CRC, n = 123) were collected from the Sun Yat-sen University Cancer Center as we previously described [20]. This study was approved by the Ethics Committee of the Ethics Committee of Sun Yat-sen University Cancer Center (No. GZR2012–123). Oral consent was obtained from patients. All experiments were performed in accordance with approved guidelines and related regulations.

Female BALB/c mice (5∼6-week-old) were obtained from the Model Animal Center of Nanjing University (Nanjing, China) and were raised under pathogen-free conditions in Sun Yat-sen University (SYSU) Animal Center (Guangzhou, China). Ethical approval for the use of animals in this project was obtained from the SYSU Animal Experimentation Ethics Committee [SYSU-IACUC-2018–000066].

### Strains and growth conditions

*Fusobacterium nucleatum* (ATCC 25586) and *Bacteroides fragilis* (*B*. *fragilis*, ATCC 25285) have been maintained in our laboratory and revived on blood agar plate (Huankai, China) under anaerobic conditions by using AnaeroPack (Mitsubishi Gas Chemical Co., Japan) for 48–72 h at 37°C. Bacteria harvested from the plates were suspended in DMEM diluted in BHI, and then cultured in a liquid medium at 37°C for 48 h under anaerobic conditions.

### Bioinformatics analysis

The 3D structures of proteins were obtained from the Protein Data Bank (PDB), [21] while possible structure models were taken from ModBase (https://modbase.compbio.ucsf.edu/modbase-cgi/index.cgi). [22] The phylogeny tree was constructed using the neighbor-joining (N-J) method in MEGA6.0 software. The antigenic index plots of Dps protein were predicted using the bioinformatics predicted antigenic peptides (BPAP) system (http://imed.med.ucm.es/Tools/antigenic.pl) [23].

### Protein expression and purification

PCR amplified the Fn-Dps gene using primers F1 and R1 (**S1 Table**) and Fn ATCC 25586 DNA as templates. The PCR products were digested with *Nco* I and an *Xho* I and ligated into pET28a (+) expression vector (Novagen, USA). The recombinant plasmids of pET28a-Fn-Dps were transformed into *E*. *coli* BL21 (DE3). The expression of the recombinant Fn-Dps was induced with 0.8 mM isopropyl β-d-1-thiogalactopyranoside (IPTG) for continuous 16 h cultivation at 25°C. The recombinant Fn-Dps with a His tag was purified using a Ni-NTA column. (Sangon Biotech, China). The endotoxin in Fn-Dps was removed using the Endotoxin Removal Kit (GenScript, China).

The total bacterial proteins from IPTG-induced *E*. *coli* BL21 harboring pET28a (+) empty vector were removed endotoxin and then quantified and used as a control protein. The protein concentration was determined with a BCA Kit (Beyotime, China).

### Protein identification by Nano LC-MS/MS

Protein identification by Nano LC-MS/MS was performed as described previously.[15] The recombinant Fn-Dps was obtained by in-gel digestion of selected protein bands in silver-stained SDS-PAGE gels, and then digested with trypsin (Sigma, USA). Fn culture media were collected, freeze-dried, and trypsin-digested. Subsequently, the tryptic protein hydrolysates were analyzed using Nano LC-Q Exactive Plus MS (Thermo Fisher, USA). Proteins were identified using Mascot software (Version 2.2.1, Matrix Science, UK) to search the Swiss-Prot protein database (http://www.expasy.ch/sprot/).

### Circular dichroism spectroscopy analysis

Fn-Dps (1.0 μM) was mixed with or without FeSO_4_ (40, 120, 200, 400 μM) for 15 min. Circular dichroism spectra were measured using a Chirascan spectrometer (Applied Photophysics, Ltd., UK). Circular dichroism spectra settings were as follows: wavelength 195–260 nm, step size 1 nm, bandwidth 1 nm, scan rate 50 nm/min, and response time 0.5 s. Each sample was repeatedly measured three times, and the mean value was calculated.

### Cell culture

The THP1, RAW264.7, J774A.1, RKO, and CT26 cell lines were purchased from the Chinese Academy of Sciences. RAW264.7 and J774A.1 are murine macrophage cell lines. THP1 are human macrophage cell lines. RKO cell lines are human colorectal carcinoma cell lines. CT26 cell lines are murine colon adenocarcinoma cell lines. To obtain THP1 macrophages (M-THP-1), THP1 monocytes were differentiated with 320 nM of PMA (Invivogen, USA) for 24 h. All these cells were cultured in RPMI 1640 medium (Gibco, USA) supplemented with 10% fetal bovine serum (FBS,Gibco, USA) under a humidified 5% CO_2_ atmosphere at 37°C.

### Erythrocytes isolation and staining

Whole blood (Na2EDTA as an anticoagulant, 1.5 mg/ml) was taken from mice, and leukocyte-depleted erythrocytes were isolated by gentle centrifugation (2,000g, 15 min). The erythrocytes were transferred to the RPMI 1640 medium (Gibco, USA) containing 10% FBS (Gibco, USA); They were then preserved in a CO_2_ incubator set at 37°C with 5% CO_2_. 5× 10^10^ erythrocytes were incubated with PBS (control), Fn-Dps (0.5, 1.0, 2.0, 10 μM) with or without FeSO_4_ (Fe^2+^, 10 μM) for the indicated times. Erythrocytes staining was performed using the Wright-Giemsa stain kit (Solarbio, China) according to the manufacturer’s instructions.

### Cell viability assay

Cell viability was measured by Cell Counting Kit-8 (CCK-8, Fude Biotech, China**)** following the manufacturer’s instructions. In brief, cells were plated into 96-well plates at a density of 5.0 ×10^3^ cells/well. Then, cells were treated with Fn-Dps for the indicated times (0, 24, 48, and 72 h), followed by a treatment of 10 μl CCK-8 for 2 h at 37°C. The absorbance was measured at 450 nm using a microplate reader (Bio-Rad, USA).

### RNA sequencing and RT-qPCR

Cell samples were sent to IGE Biotechnology Ltd. (Guangzhou, China) for transcriptome sequencing. The experimental procedures, including library preparation and sequencing, were the standard procedures provided by Illumina. Sequencing was performed with an Illumina HiSeq 3000 system (Illumina, USA).

Total RNA was extracted using Trizol (Invitrogen, USA) with on-column DNase I (Sigma, USA) digestion following the manufacturer’s protocol. The first-strand cDNA was synthesized using an iScript cDNA Synthesis kit (Bio-Rad, USA). RT-qPCR was performed on Roche LightCycler 96 System (Roche, Germany) using Fast SYBR Green Master Mix (Takara, Japan). GAPDH was used as reference genes for relative quantification. The primers used in the real-time PCR are presented in **S1 Table.** The 2^-ΔΔCt^ method was utilized to quantify gene expression.

### Transfection with small interfering RNA (siRNA)

Transfection of siRNA was carried out using lipofectamine 3000 (Invitrogen, USA), according to the manufacturer’s instructions. CCL2-siRNA (si-CCL2), CCL7-siRNA (si-CCL7), and negative control-siRNA (si-NC) were purchased from GenePharma (GenePharma, China). Sequences of siRNAs are listed in **S1 Table**.

### Immunofluorescence staining

Fn invaded into macrophages and then was assessed as described [16]. Briefly, rabbit anti-Fn polyclonal antibody (homemade, 1:5,000), mouse anti-Fn-Dps polyclonal antibody (homemade, 1:1,000), or α-tubulin antibody (1:200, Bioworld, USA) were incubated overnight at 4°C. Then, incubate with anti-rabbit IgG DyLight 594 (1:100, Abcam, USA), and anti-mouse IgG DyLight 488 (1:100 Abcam, USA) fluorescent secondary antibodies at room temperature for 45 minutes. DAPI (Thermo Fisher, USA) was used to stain the nuclei for 5 min. Images were acquired with a laser scanning confocal microscope (LSCM, Olympus, FV3000).

### Intracellular survival assays

Bacterial infection for intracellular entry and proliferation was assessed as we previously described [16]. Briefly, RAW264.7 cells were infected with live Fn or heat-killed Fn (K-Fn) at a multiplicity of infection (MOI) of 10:1 at 37°C with 5% CO_2_ for 12 h. Then, Fn-infected cells were washed three times with PBS, followed by gentamicin incubation (100 μg/ml) for 2 h to remove extracellular bacteria. Ultimately, cells were lysed with 0.3% Triton X-100. The released intracellular Fn were enumerated as colony-forming units (CFU) by plating on blood agar plate (Huankai, China) at 37°C for 48 h under anaerobic conditions. *B*. *fragilis* was used as a control.

### Immunization and assessment of protective efficacy

For systemic immunization, BALB/c mice were immunized by subcutaneous (*s*.*c*.) injection of aluminum hydroxide (Alum, 2 mg, Sigma, USA), Fn-Dps (100 μg) or Fn-Dps (100 μg) combined with alum one week apart. For mucosal immunization, mice were immunized by intragastric (*i*.*g*.) administration of cholera toxin B subunit (CTB, 10 μg, Sigma, USA), Fn-Dps (100 μg) or Fn-Dps (100 μg) combined with CTB. Mice were treated with PBS as control group. One week after the final immunization, mice were challenged *i*.*g*. with 1.0 ×10^8^ Fn once daily for 7 days. The number of colonizing Fn in mouse intestinal tissue was determined by qPCR according to a previous study [23].

### ELISA

The concentrations of CCL2/CCL7 in the conditioned media were measured by ELISA kits (R&D Systems Europe Ltd, UK) following the manufacturer’s instructions. The levels of anti-Fn-Dps were measured by ELISA. ELISA plates were coated with Fn-Dps (0.5 μg/ml) overnight at 4°C. Sera from the immunized mice were diluted from 1:1,000 to 1:4,096,000. Colon mucus was collected from a 5.0 cm portion of the colon intestine and diluted at 1:10 to 20480. Human serum samples were diluted at 1:1,000 to assay anti-Fn-Dps IgG and diluted at 1:100 to assay anti- Fn-Dps IgA. HRP-goat anti-mouse/human IgG or IgA (1:5,000, EarthOx, USA) was used as a secondary antibody. The chromogenic substrate (tetramethylbenzidine) solution was used for HRP detection and was terminated by 2M H_2_SO_4._ The OD values at 450 nm were read by ELISA spectrophotometer (Bio-Rad, USA).

### Western-blot analysis

Cells were lysed in RIPA buffer and boiled for 10 min. Total cell proteins were loaded onto a 10% SDS-PAGE, proteins were transferred from the gel to a PVDF membrane. The membrane was blocked with 5% skim milk powder for 2 h. Subsequently, the membranes were incubated overnight with E-cadherin, N-cadherin, Vimentin (1:2,000, Abcam), and Snail (1:2,000, CST) antibodies after being blocked for 1 h. After washed in PBST, the membranes were incubated with horseradish peroxidase-conjugated (HRP) secondary antibody (1:5,000, EarthOx Life Sciences, USA) for 1 h on shake at room temperature. GAPDH protein levels were determined using a specific antibody (1:5,000, Bioworld, USA) as a loading control. Protein bands were visualized with an enhanced chemiluminescence detection system (Tianneng, China).

For human serum samples, recombinant Fn-Dps were separated by 15% SDS-PAGE, and the membranes were incubated with serum from 8 healthy subjects and 8 Fn-high CRC individuals as primary antibodies. Goat anti-human IgG-HRP or goat anti-human IgA-HRP (1:5,000, BOSTER, China) was used as a secondary antibody.

### Transwell migration assay

Transwell inserts (8.0 μm pore size, Corning) in 24-well plates were used. 1.0 × 10^5^ CT26 or RKO cells in 100 μl serum-free RPMI 1640 were added to the upper chambers, and the lower chamber was added 600 μl medium with 1.0 × 10^5^ RAW264.7 cells or J774A.1 cells or M-Thp-1 cells treated with PBS (control), Fn-Dps (1.0 μM), CCL2 (20 ng/ml, BioLegend, USA), CCL7(20 ng/ml, BioLegend, USA), CCL2+CCL7 (20 ng/ml), and Fn-Dps (1.0 μM) combined with CCL2 + CCL7 neutralizing antibody (CCL2/7 nAb, 1.5 μg/ml, R&D Systems, USA) to serve as a chemotactic agent.

After 24 h, cells across pores were fixed with 4% paraformaldehyde and stained with 1% crystal violet solution. Five fields were randomly chosen for each chamber, and cells were counted. The migration was analyzed by ImageJ. Each Transwell assay was repeated in three independent experiments.

### Wound-healing assay

CT26 or RKO cells were seeded in 60-mm dishes to create a confluent monolayer. Then, the cell monolayer was scratched in a straight line using a 10 μl pipette tip. CT26 or RKO cells were incubated with 2 ml of conditioned media (CM) to perform the *in vitro* scratch assay. CM was collected from RAW264.7, J774A.1, and M-Thp-1 cells treated for 48h with PBS (control), Fn-Dps (1.0 μM) CCL2 (20 ng/ml, BioLegend, USA), CCL7 (20 ng/ml, BioLegend, USA), CCL2+CCL7 (20 ng/ml), and Fn-Dps (1.0 μM) combined with CCL2 + CCL7 neutralizing antibody (CCL2/7 nAb, 1.5 μg/ml, R&D Systems, USA). Images were taken at 0 and 48 h after scratching. The migration distance was measured using ImageJ software.

### Colon cancer metastasis mouse model

For the lung metastases model, 35 female BALB/c mice were randomly divided into 7 groups (5 mice in each group). 1.0×10^6^ CT26 cells were injected intravenously through the tail vein (*i*.*v*.) of each mouse. Three days later, mice were injected intravenously Fn (10^8^ CFU), Fn-Dps (1.0 μM), Fn-Dps (10 μM), Fn-Dps (1.0 μM) combined with CCL2/7 nAb (2 mg/kg, R&D Systems, USA), Fn-Dps (10 μM) combined with CCL2/7 nAb (2 mg/kg), PBS (control1 and control 2) every two days for a week.

For the liver metastasis model, 35 female BALB/c mice were randomly divided into 7 groups (5 mice in each group). Mice were anesthetized using intraperitoneal injection of pentobarbital sodium (Tocris, China). Under aseptic conditions, a small longitudinal incision was made in the left upper flank to visualize the spleen, and 2.0×10^6^ CT26 cells were injected under the spleen capsule. Three days later, mice were treated in the same way as with lung metastases model.

Mice were sacrificed 2 weeks after injection, and the livers and lungs were surgically excised. The number and size of metastatic foci in the liver and lungs were documented.

### Histology

Tissues were fixed in 10% buffered formalin for 24 h, processed, and embedded in paraffin for sectioning according to conventional methods. The paraffin-embedded tissues were then cut into 5.0-μm thick sections and stained with hematoxylin and eosin (H&E). For immunohistochemical staining (IHC), tissue sections were incubated with primary antibodies against CCL2 and CCL7 (1:200, Affinity, USA), N-cadherin and vimentin (1:200, Abcam, USA) and HIF-1 (1:200, CST, USA) overnight at 4°C. After washing with PBST, the sections were incubated with an HRP-conjugated anti-mouse or anti-rabbit secondary antibody (1:5,000, CST, USA) for 1 h at room temperature. The sections were developed with 3-diaminobenzidine tetrahydrochloride for 10 s, which was followed by counterstaining with 10% Mayer’s hematoxylin for 4 min.

### Statistical analysis

The data represent the mean ± SD unless otherwise indicated. Data were analyzed by two-tailed unpaired Student’s t-test between two groups and by one-way ANOVA followed by Bonferroni test for multiple comparisons. Significance was considered *P* < 0.05. Statistical analyses were performed using GraphPad 8.0.

## Results

### Identification of Fn-Dps protein in the culture supernatant of Fn under nutrient starvation

To explore the secreted or soluble protein of Fn, the organism culture media were collected after Fn was anaerobically grown at 37°C for 48 h in BHI or 1640–diluted BHI. The lowest limited concentration of BHI for the survival of starved Fn was 0.25% BHI in 99.75% 1640 medium. The soluble protein of the culture supernatant of Fn was identified by nano-LC–MS/MS. As shown in **S2 Table**, 18 high abundance proteins were found in the culture supernatant of Fn under starved conditions, in which Fn1079 (Q8REM0) exhibited the highest peptide abundance among these proteins. Interestingly, a moderate abundance of Fn1079 was also detected by MS under the nutrient-rich growth condition using undiluted BHI media **(S3 Table)**.

**Fig 1:**
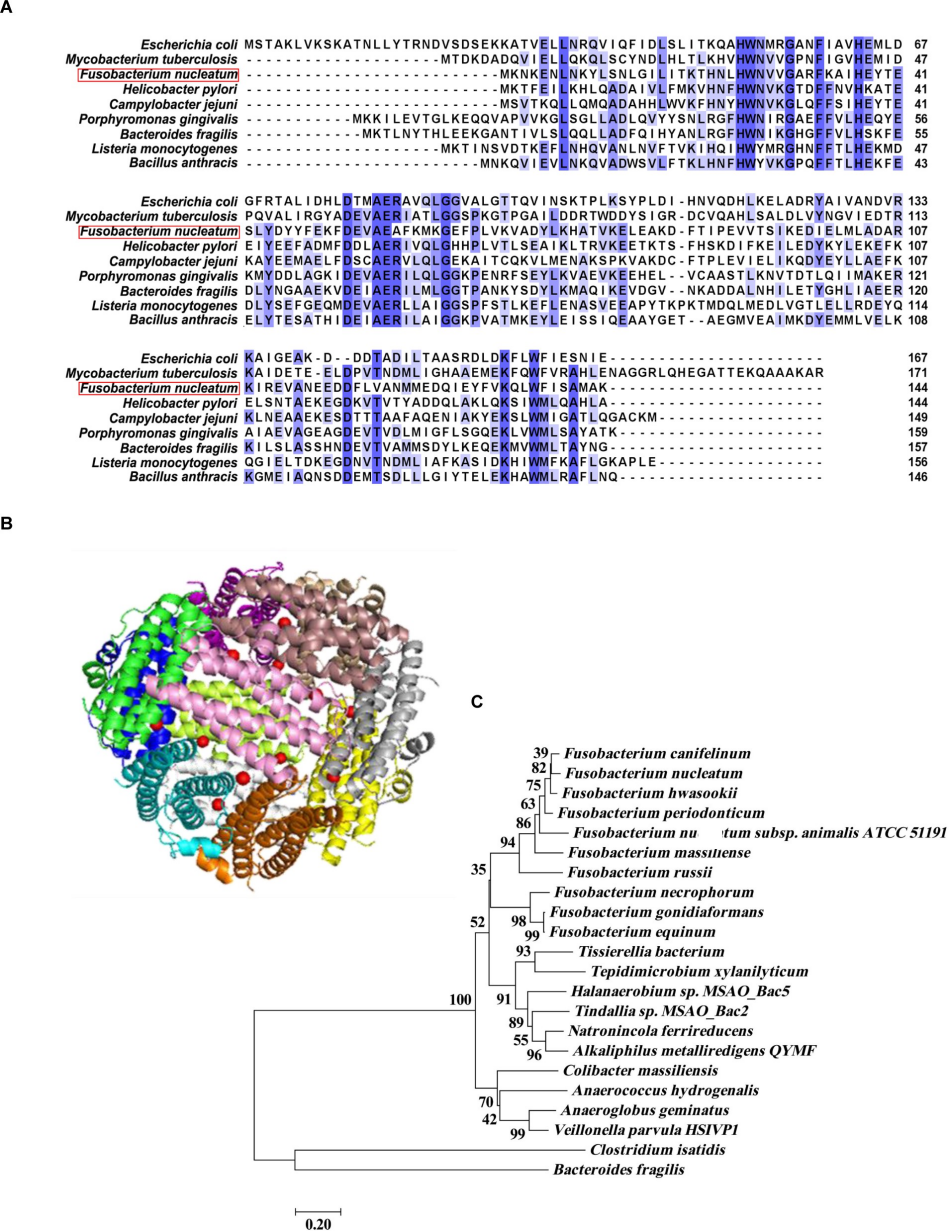
Bioinformatics analysis of protein Fn-Dps. (A) Alignment of the amino acid sequence of Fn1079 (Fn-Dps) with those of Dps previously isolated and determined in other prokaryotes. (B) 3D Fn-Dps protein structure obtained from ModBase server analysis. (C) The phylogenetic tree of Fn-Dps (Q8REM0) was constructed by MEGA 5.1.

Then, the conserved domains prediction by the NCBI Conserved Domains Database showed that Fn1079 (Fn-Dps) belongs to a member of the DNA-binding ferritin-like protein (Dps) family **(S1 Fig)**. Moreover, the 144 amino acid sequences of Fn-Dps were aligned using CLUSTALW software **(Fig 1A)**. Fn-Dps can form a homo-dodecamer with twelve ferrous ion ligands using ModBase analysis **(Fig 1B)**.

BLASTP analysis revealed that Fn-Dps has no homology with known eukaryote species and only showed a low percentage identity with other obligate anaerobe bacteria (<50%) except the genus *Fusobacterium* **(S4 and S5 Tables)**. Fn-Dps exhibited only approximately 30% or less identity with nonobligated anaerobes, such as the *Helicobacter pylori* (Hp)-Nap protein **(S5 Table)**. Next, the phylogenetic tree was constructed based on the alignment of Fn-Dps amino acid sequences (GI: 19714672), and the results showed an evolutionary relationship between Fn and other species **(Fig 1C).**

### Recombinant Fn-Dps is a dodecamer and causes erythrocytes damage *in vitro*

**Fig 2:**
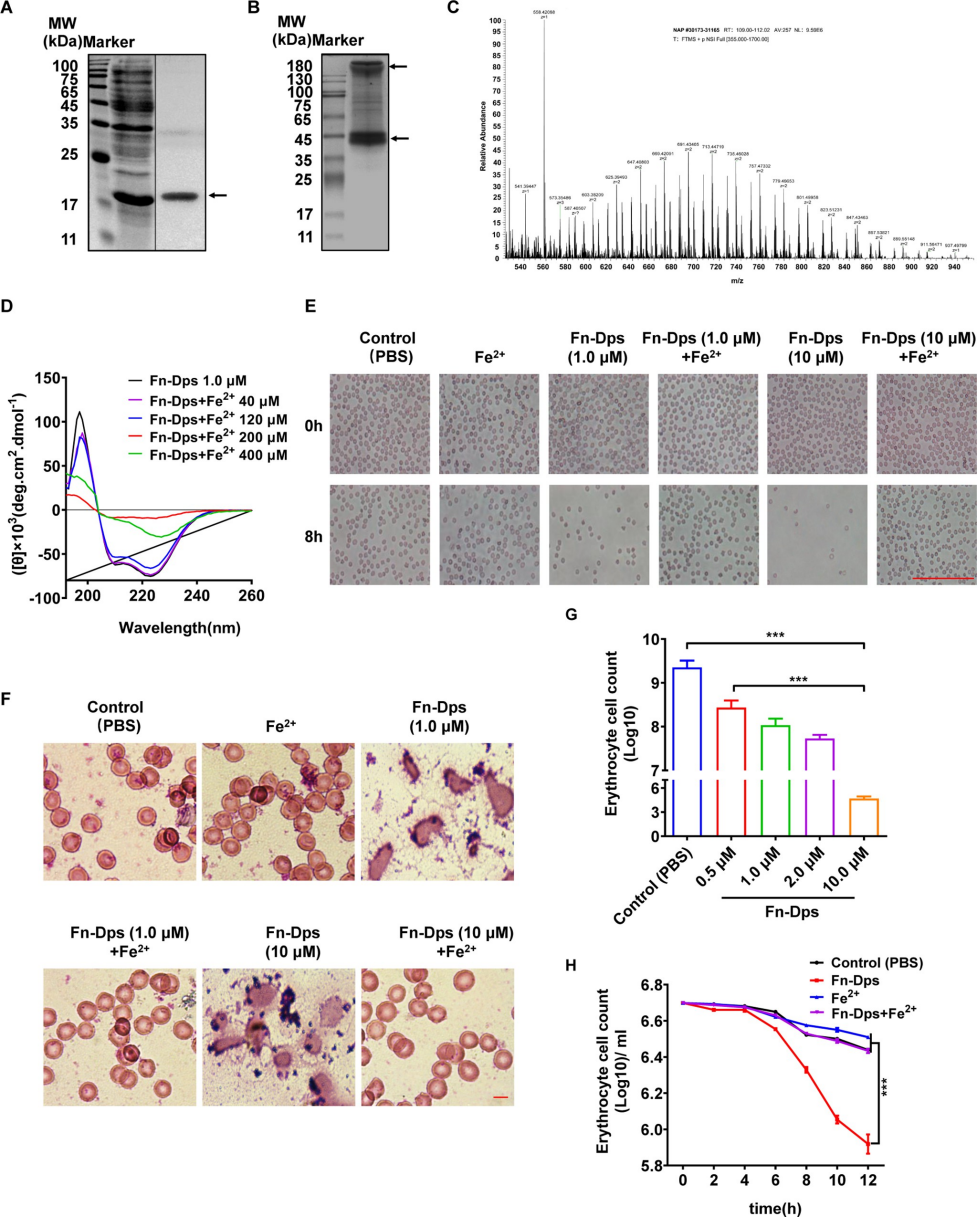
Preparation of recombinant Fn-Dps. (A) Fn-Dps with a His tag was purified using a Ni-NTA column. (B) The Fn-Dps protein polymer was determined using NATIVE-PAGE. (C) Identification of Fn-Dps by using nano LC–MS/MS. (D) Fn-Dps polymerization morphological changes during FeSO_4_ (40, 120, 200, 400 μM) interactions by circular dichroism (CD) spectroscopy. (E) Morphological changes in mouse erythrocytes after 8 h posttreatment with Fn-Dps (1.0, 10 μM) with or without FeSO4 (Fe^2+^,10 μM), (F) Erythrocytes were stained with Wright’s staining solution in mouse erythrocytes after 8 h posttreatment with Fn-Dps (1.0, 10 μM) with or without FeSO4 (Fe^2+^,10 μM). (G) Erythrocytes were treated with with PBS (control), Fn-Dps 0.5 μM, 1.0 μM, 2.0 μM, 10 μM) for 24 h and then counted under a microscope. (H) Erythrocytes were counted under a microscope following treatment with PBS (control), Fn-Dps (1.0 μM), FeSO_4_ (10 μM), or Fn-Dps (1.0 μM)+FeSO_4_ (10 μM) for the indicated times. Data are expressed as mean ± SD and compared by Student’s t test (G) or One-way ANOVA (H). ***P <0.001. n = 3 independent experiments.

Next, the pET28a-Fn-Dps plasmid was constructed to prepare Fn-Dps recombinant protein. SDS-PAGE analysis showed a marked increase in the expression of a soluble protein in *E*. *coli* BL21 harboring pET28a-Fn-Dps after IPTG induction **(Figs S2 and 2A)**. Then, the oligomeric state of the purified recombinant Fn-Dps was examined by native PAGE. As shown in **Fig 2B**, two proteins with apparent molecular weights of approximately 200 kDa and 50 kDa were present in the native gel, indicating that recombinant Fn-Dps was expressed as a soluble multimeric protein in *E*. *coli*. Compared with the 17 kDa monomer on SDS-PAGE, molecular weights of approximately 200 kDa and 50 kDa on the native protein indicated that the Fn-Dps protein was a dodecamer (formed *via* four trimmers) with identical subunits. The protein with molecular weight of approximately 17 kDa was purified using a Ni-NTA agarose column and then validated as Fn-Nap (Fn-Dps) using nano-LC-MS/MS analysis **(Fig 2C)**.

Based on the properties of the ferritin-like protein, Fn-Dps was measured to test the polymerization morphological changes during iron interaction by circular dichroism (CD) spectroscopy. The CD spectrum of Fn-Dps was characterized by a positive peak at approximately 197 nm with two negative peaks between 210 nm and 240 nm, a typical feature of the overwhelming content of α-helices **(Fig 2D)**. With the addition of a high concentration of ferrous ions (Fe^2+^, 400 μM), the absorption peak at approximately 197 nm decreased sharply, while the absorption peaks between 210 nm and 240 nm increased, indicating that the structure of Fn-Dps changed during the Fe^2+^ interaction **(Fig 2D)**. These results identified that Fn-Dps is a dodecameric protein consisting of 17 kDa monomers with a central cavity where ferrous ions bind.

Next, we investigated the cytotoxic effects of Fn-Dps with/without Fe^2+^ on erythrocytes. As shown in **Figs 2E and 2F and S3**, erythrocytes were lysed and destroyed obviously, and their number was reduced at 8 h under Fn-Dps and Fn treatment, whereas no visible morphological change was observed in the control and Fe^2+^-treated groups. However, Fe^2+^ supplementation restored the deformed erythrocytes to their normal shape. Moreover, Fn-Dps exhibited high cytotoxic activity against erythrocytes in a concentration-dependent manner (**Fig 2G**). When treated with a relatively low dose of Fn-Dps (0.5 μM), a reduction in the count was observed in a time-dependent manner, and the inhibition rate was over 80% in Fn-Dps-treated cells at 8 h post-incubation (**Fig 2H**).

### Identifying Fn-Dps-induced differentially expressed genes in RAW264.7 cells

**Fig 3:**
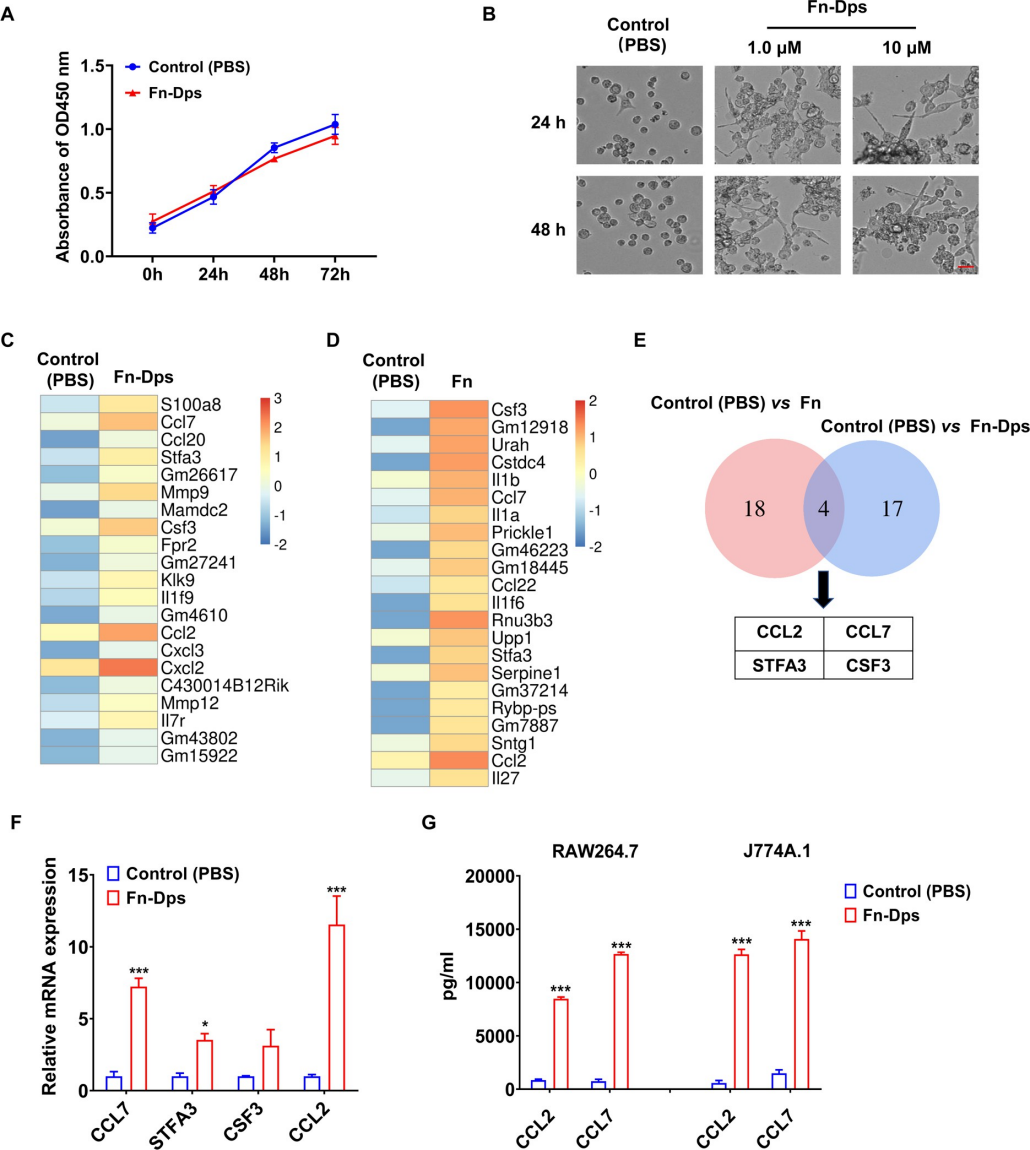
Fn-Dps stimulated the expression of CCL2/CCL7 in RAW264.7 cells. (A) The CCK-8 assay was used to detect the effects of Fn-Dps (1.0 μM) on RAW264.7 cell proliferation at the indicated times. (B) Morphological changes of RAW264.7 cells stimulated by Fn-Dps (1.0, 10 μM). Scale bar  =  400 μm. (C) Heatmap based on the top 21 upregulated miRNAs between PBS-treated RAW264.7 cells (control) and 1.0 μM Fn-Dps-treated RAW264.7 cells. (red, upregulation; blue, downregulation). (D) Heatmap based on the top 22 upregulated miRNAs between PBS-treated RAW264.7 cells (control) and Fn-infected RAW264.7 cells. (red: upregulation, blue: downregulation). (E) Venn diagram illustrating the relationship between differentially expressed mRNAs in the two discrete comparisons: Control *vs*. Fn (red circle) and Control *vs*. Fn-Dps (blue circle). (F) Differentially expressed genes were validated by RT–qPCR. (G) ELISA assay detecting CCL2/CCL7 levels after1.0 μM Fn-Dps-treated RAW264.7 and J774A.1 cells. Scale bar  =  100 μm. Data are expressed as mean ± SD and compared by One-way ANOVA test (A) or Student’s t test (F and G). *P<0.05, ***P <0.001. n = 3 independent experiments.

To assess the possible effects of Fn-Dps on RAW264.7 and J774A.1 cell viability, we performed a CCK8 assay. The results revealed that there was no significant difference in RAW264.7 and J774A.1 cells viability whether they were treated with either Fn-Dps or not **(Figs 3A and S4)**. However, RAW264.7 and J774A.1 cells exhibited obvious morphological changes into macrophage-like branching and elongation shapes when treated with Fn-Dps *in vitro* (**Figs 3B and S5**).

We performed two independent transcriptome profiling experiments in Fn-Dps-treated and Fn-infected RAW264.7 cells. Based on log2 fold change > 2 and adjusted *p*-value < 0.05 criteria. In Fn-Dps treatment group, we identified 408 deregulated genes (307 upregulated and 101 downregulated). In Fn treatment group, we identified 3155 deregulated genes (1414 upregulated and 1741 downregulated). The heatmap was constructed indicating differential gene expression levels of all genes (**S6 Fig**). To understand the function of those different genes, Gene Ontology (GO) and KEGG pathway enrichment were analyzed **(S7 and S8 Figs)**. The top 21 upregulated genes (log2 fold change > 4, *p*-value < 0.05) in Fn-Dps-treated RAW264.7 cells are presented in **Fig 3C**, and the top 22 upregulated genes in Fn-infected RAW264.7 cells are presented in **Fig 3D**. Next, we identified 4 upregulated overlapping genes (colony stimulating factor 3, CSF3; chemokines CCL2, chemokines CCL7, and Stefin A3, STFA3) in Fn-Dps-treated and Fn-infected RAW264.7 cells **(Fig 3E).** Among them, RT-qPCR analysis indicated that CCL2/7 were significantly increased after Fn-Dps treatment in RAW264.7 cells **(Fig 3F)**. Moreover, CCL2/7 were significantly increased after infection with Fn but not with *B*. *fragilis* control **(S9 Fig**). Furthermore, ELISA also showed the elevated level of CCL2/7 in both RAW264.7 and J774A.1 cells after stimulated by Fn-Dps **(Fig 3G)**.

### Fn-Dps increases the intracellular survival of Fn in RAW264.7 cells

**Fig 4:**
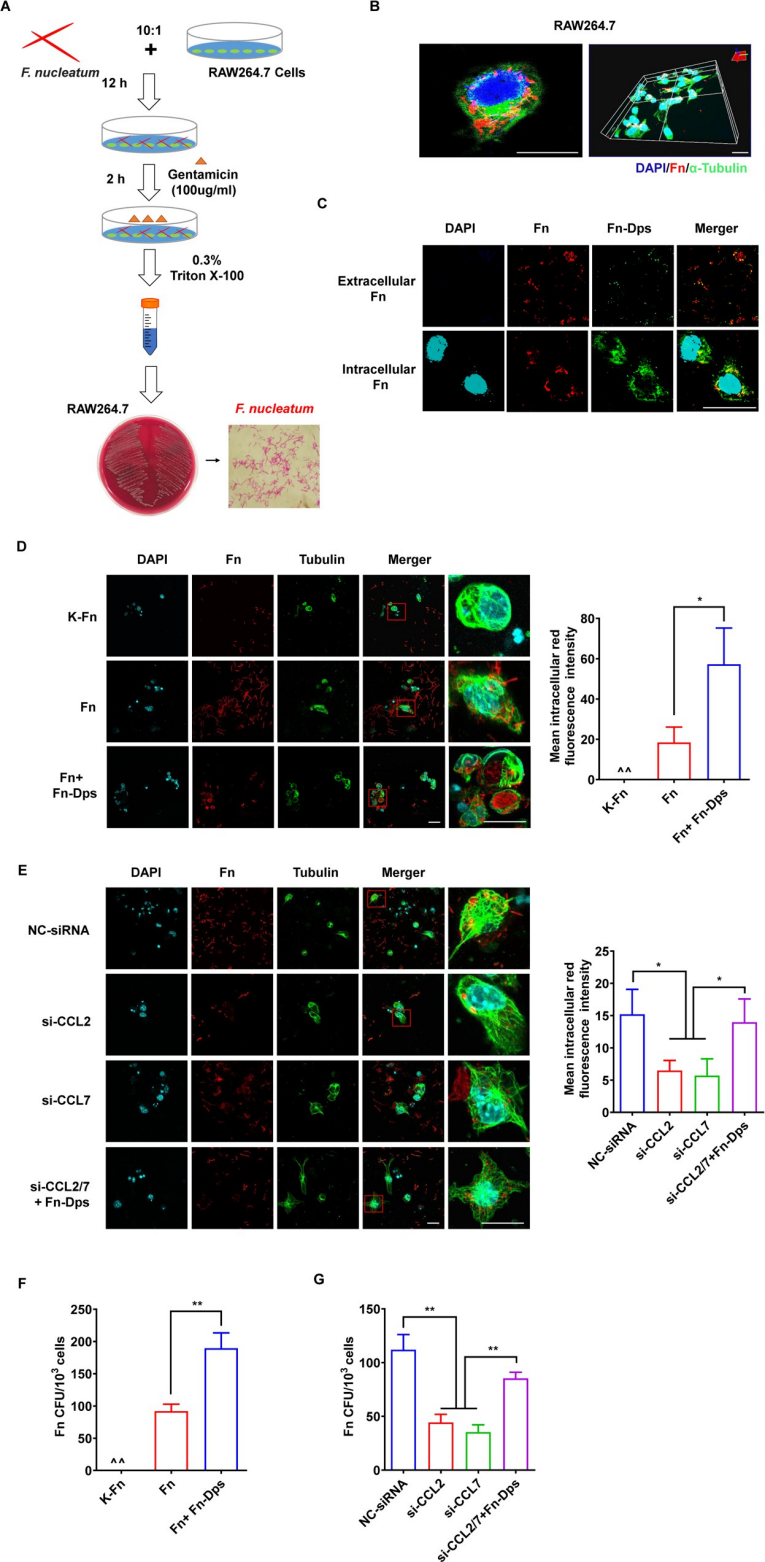
Fn-Dps enhances the intracellular survival of Fn in macrophages. RAW264.7 cells were infected with Fn or heat-killed Fn (K-Fn) at an MOI of 10:1 (bacteria: cells). (A) Intracellular and extracellular Fn-Dps levels in RAW264.7 cells. (B) RAW264.7 cells were infected with K-Fn, Fn, or Fn-Dps (1.0 μM) (Fn+ Fn-Dps). (C) Colony forming units (CFUs) in RAW264.7 cell lysates. (D) Fn-infected RAW264.7 cells with or without NC-siRNA (50 nM), si-CCL2 (50 nM), si-CCL7 (50 nM) or si-CCL2/7 (50 nM) + Fn-Dps (1.0 μM). (E) Colony forming units (CFUs) in RAW264.7 cell lysates. Scale bar  =  50 μm. Data are expressed as mean ± SD and compared by Student’s t test (C and E). *P<0.05, **P<0.01. n = 3 independent experiments.

To further investigate whether Fn-Dps are involved in the intracellular survival of Fn. RAW264.7 macrophages were treated with Fn and then were incubated with 100 μg/ml of gentamycin to kill extracellular Fn. Based on MICs, Fn ATCC 25586 is susceptible to gentamicin (MIC 4 μg/ml) **(S10 Fig)**. The schematic illustration of cells–bacteria co-culture experiment is shown in **Fig 4A**. Intracellular localization of Fn was observed by confocal laser scanning microscopy (LSCM) **(Fig 4B)**. Intracellular Fn showed strong Fn-Dps staining, whereas extracellular Fn staining was either weak or absent. Moreover, LSCM revealed that Fn was present in the RAW264.7 cells at 12 h post-infection, whereas heat-killed Fn (K-Fn) was not observed to enter host cells (**Fig 4C)**. Remarkably, a higher number of intracellular Fn was observed in the Fn-Dps-treated group than in the untreated group **(Fig 4D)**. Furthermore, a significantly reduced intracellular Fn was observed in the si-CCL2/7 group compared with the negative control siRNA (NC-siRNA) group at 24 h post-infection, whereas the amount of intracellular Fn was partially restored in the Fn-Dps-treated si-CCL2/7 group (**Fig 4E**).

Moreover, Fn-infected RAW264.7 cells were taken from the co-culture dish and prepared for viable counts of intracellular survival of Fn after gentamycin killing of extracellular bacteria. The results of viable counts were consistent with the immunofluorescence observations (**Fig 4F and 4G**). Besides, the control bacteria *B*. *fragilis* could not survive in RAW264.7 cells with or without Fn-Dps treatment **(S11 Fig)**. These results indicated that Fn-Dps significantly increased the invasion and survival of Fn in RAW264.7 cells by upregulating the expression of CCL2/7.

### Fn-Dps induces EMT to promote the migration of CRC cells

**Fig 5:**
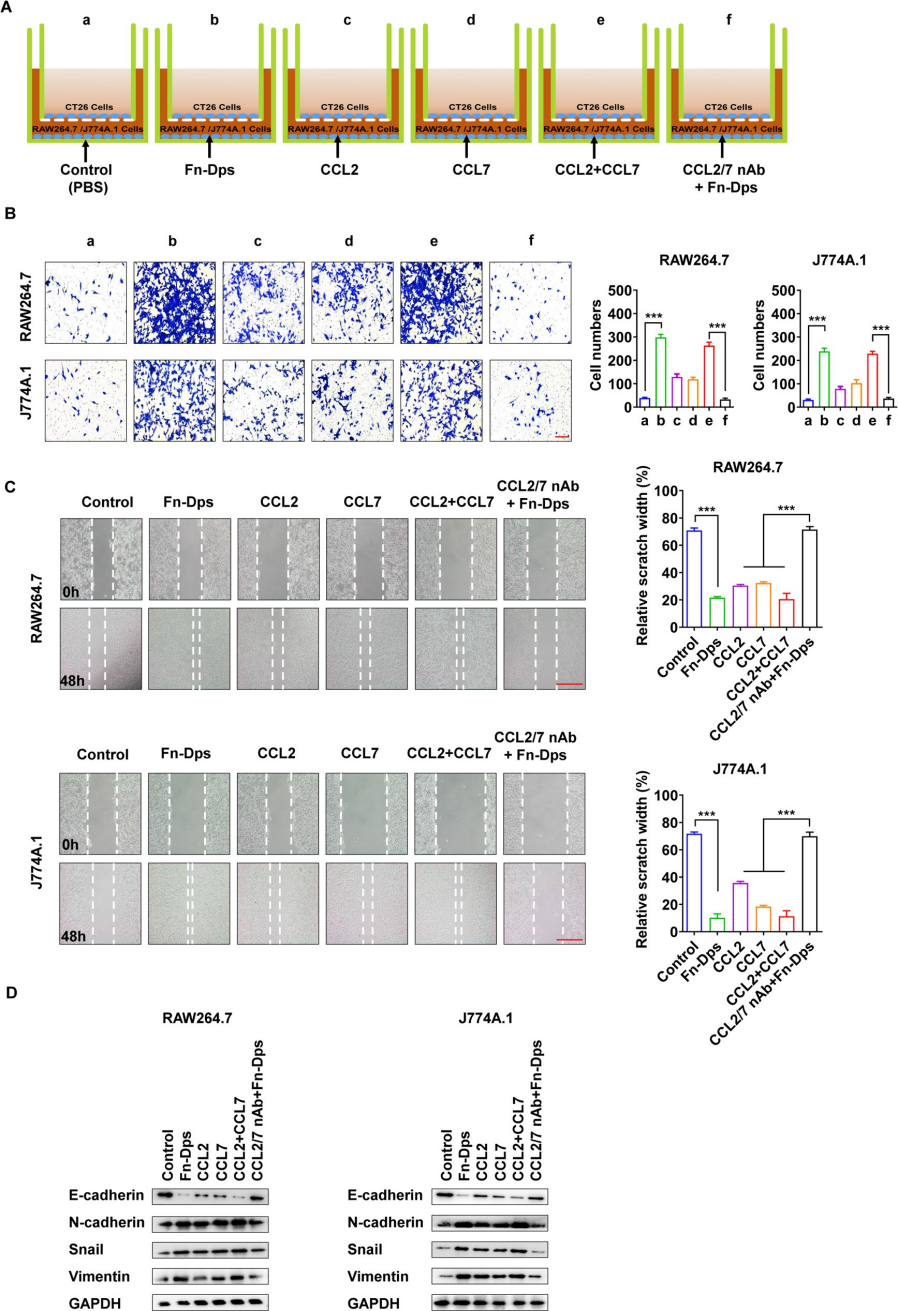
Fn-Dps promotes the migration of CT26 cells. (A) Schematic diagram of the CT26 cells migration model: (a) CT26 cells were seeded in the top well, and RAW264.7 or J774A.1 cells were added to the lower wells treated with PBS (control), (b) or treated with Fn-Dps, (c) or treated with CCL2, (d) or treated with CCL7, (e) or treated with CCL2+CCL7, (f) or treated with CCL2/7 nAb+ Fn-Dps. Representative images of the assay (right). (B) The migration of CT26 cells was assessed using Transwell migration assays. Representative images of the assay (right). (C) Analysis of CT26 cells migration by scratch assays. CT26 cells were treated with supernatant of RAW264.7 or J774A.1 cells alone (control), with CCL2, with CCL7, with CCL2+CCL7, or with supernatant of RAW264.7 or J774A.1 cells treated with CCL2/7nAb + Fn-Dps. Scratch area was recorded after treatment for 48 h. Representative images of the assay (right). (D) CT26 cells were treated with supernatant of RAW264.7 or J774A.1 cells alone (control), with CCL2, with CCL7, with CCL2+CCL7, or with supernatant of RAW264.7 or J774A.1 cells treated with CCL2/7nAb+Fn-Dps for 48 h. The expression of E-cadherin, N-cadherin, Snail, and Vimentin was measured by Western blot analysis. Scale bar  =  200 μm. Data are expressed as mean ± SD and compared by Student’s t test (B and C). *P<0.05, **P<0.01, ***P <0.001. n = 3 independent experiments.

To determine the effect of Fn-Dps-stimulated macrophages on CRC cells, we performed co-culture experiments with CT26 cells in the top chamber and RAW264.7 /J774A.1 cells in the bottom chamber (**Fig 5A**). The results showed that the stimulation of CT26 cells with CCL2/7 or co-culture with Fn-Dps-treated RAW264.7 and J774A.1 cells significantly promoted migration in CT26 cells compared with untreated cells. In addition, treatment with CCL2/7 neutralizing antibody (CCL2/7 nAb) significantly inhibited the migration ability of CT26 cells cocultured with RAW264.7 and J774A.1 cells **(Fig 5B)**. The result was confirmed by wound-healing assay (**Fig 5C**). Western blotting was performed to analyze EMT markers in CT26 cells. Compared with the control, CT26 cells were stimulated with CCL2/7 or cocultured with Fn-Dps-treated RAW264.7 and J774A.1 cells, and the expression of E-cadherin was decreased, while N-cadherin, Snail, and Vimentin were upregulated **(Fig 5D)**. Moreover, human colorectal carcinoma RKO cells co-cultured with THP-1 macrophages showed similar results **(S12 Fig)**. These compelling results reveal that Fn-Dps-stimulated macrophages mediate EMT to promote migratory behaviors in CRC cells.

### Immune efficacy of the Fn-Dps antigen against Fn challenge

Based on the homology analysis and antigenic peptide prediction results, Fn-Dps possessed multiple cell antigenic determinants but few cross-reactions with other commensal bacteria **(S13 Fig and S6 Table)**.

**Fig 6:**
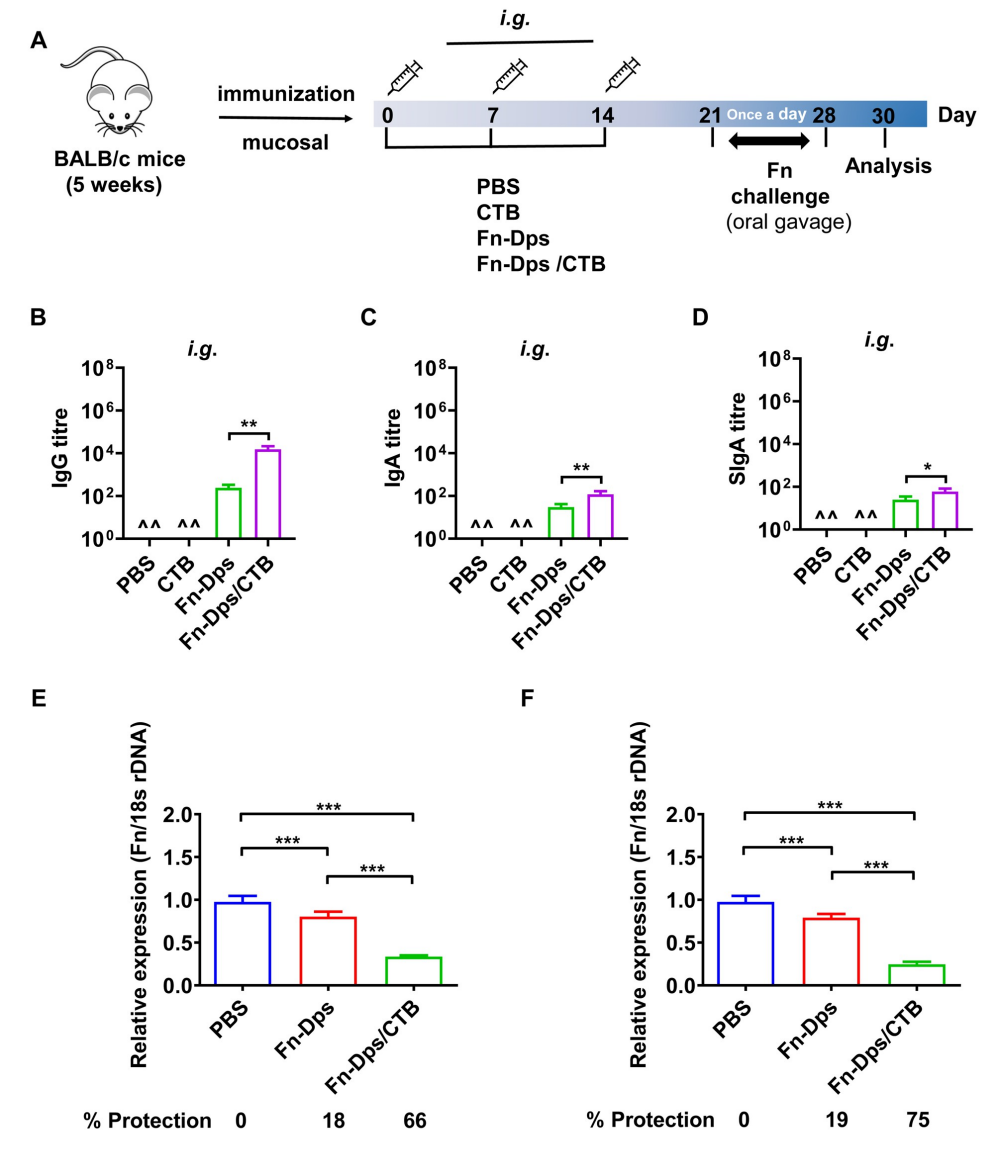
Serum antibody responses and the induction of protection against Fn by immunization with Fn-Dps. (A) Schematic of immunization experiments in mice. Mice were immunized by intragastric administration (*i*.*g*.) with PBS, cholera toxin B subunit (CTB), Fn-Dps, or Fn-Dps combined with adjuvant. (B) The anti-Fn-Dps IgG titer; (C) The anti-Fn-Dps IgA titer; (D) The anti-Fn-Dps SIgA titer. One week after the final vaccination, Fn-Dps antibody titers in sera/intestinal mucus were determined using ELISA. Colonization of Fn in the colon (E) or cecum (F) of mice perfused with stomach with PBS, Fn-Dps, or Fn-Dps combined with CTB. Colonization quantified using qPCR assay. The rate of protection was calculated using the following formula: protection rate (%) = (expression of Fn-DNA in the control group-expression of Fn*-*DNA in the immunized group)/expression of Fn-DNA in the control group × 100%. ^^represents none detected. Data are expressed as mean ± SD and compared by Student’s t test (B, C, D, E, and F). *P<0.05, **P<0.01, ***P <0.001. n = 5 independent experiments.

To investigate the immunogenicity of Fn-Dps, the mice were treated with Fn-Dps (100 μg) with or without alum/CTB by *s*.*c*. injection or *i*.*g*. administration, respectively. Mice vaccinated with either adjuvant or PBS alone served as control groups **(Figs 6A and S14A)**. As shown in **Figs 6B** and **6D and S14 and S15**, immunization with Fn-Dps, with or without adjuvant, induced a stronger immune response to the Fn-Dps antigen in *s*.*c*. injection or *i*.*g*. administration groups.

Furthermore, the mice were challenged with Fn by daily oral gavage, and the protective efficacy was determined according to the load levels of Fn-DNA within mouse intestine tissues. The results showed that mice immunized with Fn-Dps/CTB by *i*.*g*. administration obtained the highest protection rate compared with the other groups and showed a decrease in Fn load of 66% in the colon and 75% in the cecum (all *P*<0.001) **(Fig 6E and 6F**).

These findings demonstrated that Fn-Dps could elicit a strong humoral immune response even in the absence of adjuvant, but only high anti-Fn-Dps SIgA levels by mucosal immunization could confer protection against Fn in the intestinal tract.

### High levels of anti-Fn-Dps antibody were prevalent in CRC patients

**Fig 7:**
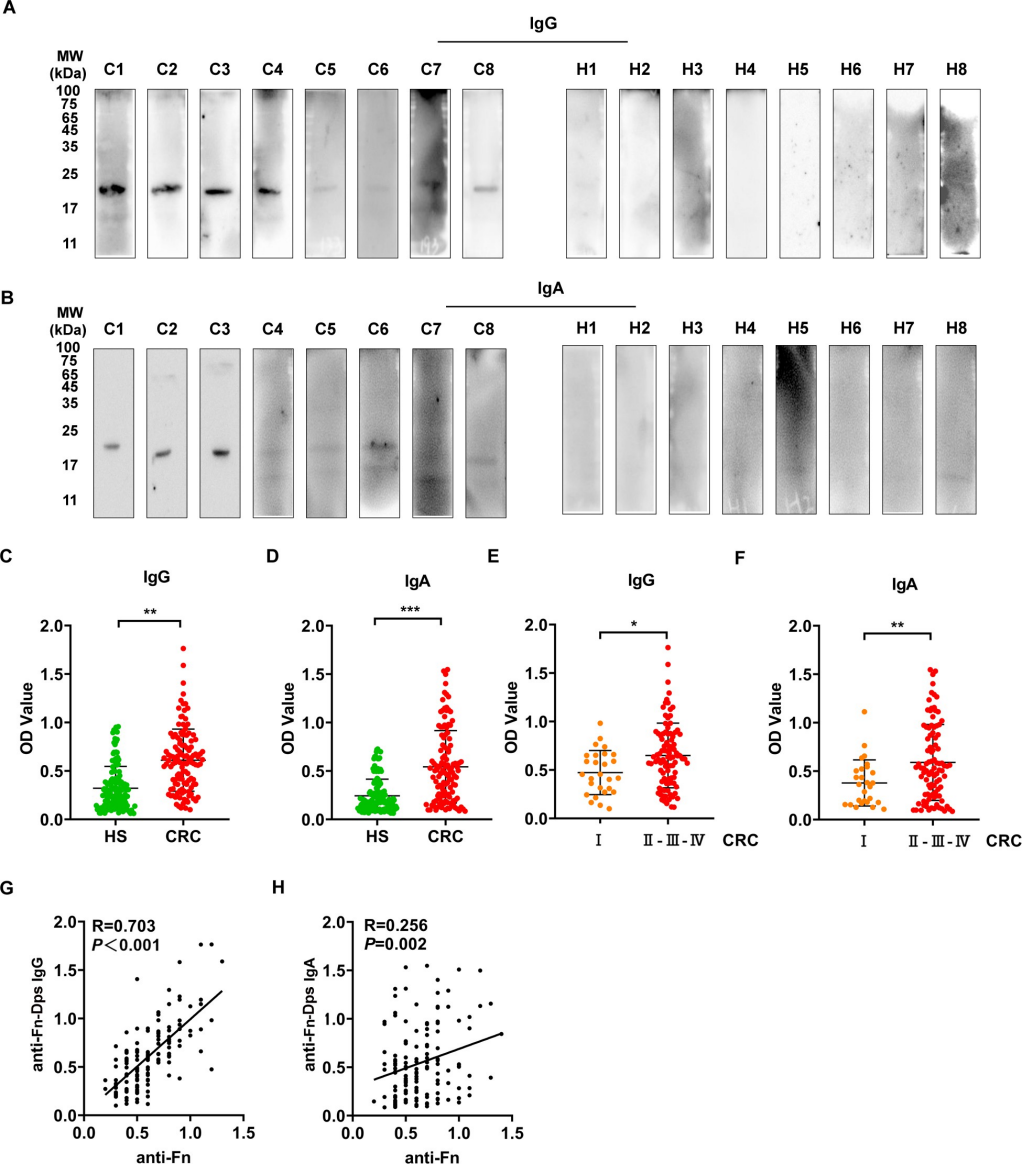
Serum anti-Fn-Dps antibody levels in CRC patients and healthy subjects. Serum anti-Fn-Dps IgG (A) and anti-Fn-Dps IgA (B) were detected by Western blotting in healthy subjects (H1–8) and Fn-positive CRC patients (C1–8). Comparison of antibody levels of anti-Fn-Dps-IgG (C) and IgA (D) in healthy subjects (HS, n = 144) and total CRC patients (n = 123). Comparison of antibody levels of anti-Fn-Dps-IgG (E) and IgA (F) in stage I CRC patients (n = 27) and stage II-IV CRC patients (n = 96). Correlation between serum anti-Fn-Dps IgG (G), anti-Fn-Dps IgA (H), and anti-Fn in CRC patients. Data are expressed as mean ± SD and compared by the Wilcoxon test (C, D, E, and F) or Pearson correlation test (G and H). *P<0.05, **P<0.01, ***P <0.001.

To further investigate the anti-Fn-Dps antibody levels in human serum samples, the specificity of the antibodies towards Fn-Dps was first verified by Western blot analysis. At the serum IgG level, Fn-Dps reacted specifically with the 10,000-fold diluted separated serum from 8 CRC patients compared with healthy subjects (HS) **(Fig 7A)**. Similar results were also observed for the anti-Fn-Dps IgA level with 1,000-fold diluted sera as primary antibodies **(Fig 7B)**.

Next, sera were used as primary antibodies and purified Fn-Dps was coated as antigens to perform the ELISA analysis. We observed that serum levels of anti-Fn-Dps IgG and IgA in the CRC patients (n = 123) were significantly higher than those in the HS (n = 144) (both *P* < 0.0001) **(Fig 7C and 7D)**. Moreover, a significant increase in anti- Fn-Dps antibody levels was detected in II-IV-stage (n = 96) when compared to I-stage CRC patients (n = 27) (both *P*<0.05) (**Fig 7E and 7F**).

The associations between anti-Fn-Dps levels and clinicopathological parameters are presented in **S7 Table**. Both anti-Fn-Dps IgG and IgA levels showed positive correlation with the clinical stage (*P* = 0.02; *P* = 0.01). In addition, Pearson’s correlation analyses showed that both the levels of anti-Fn-Dps IgG and IgA were significantly positively correlated with the antibody against Fn whole cells (anti-Fn) (R = 0.703, *P* < 0.001; R = 0.256, *P* = 0.002) (**Fig 7G and 7H**). These results confirmed that an extremely high level of serum anti-Fn-Dps antibody was prevalent in populations and that elevated anti-Fn-Dps antibody levels were present in CRC patients.

### Fn-Dps promotes CRC metastasis *in vivo*

**Fig 8:**
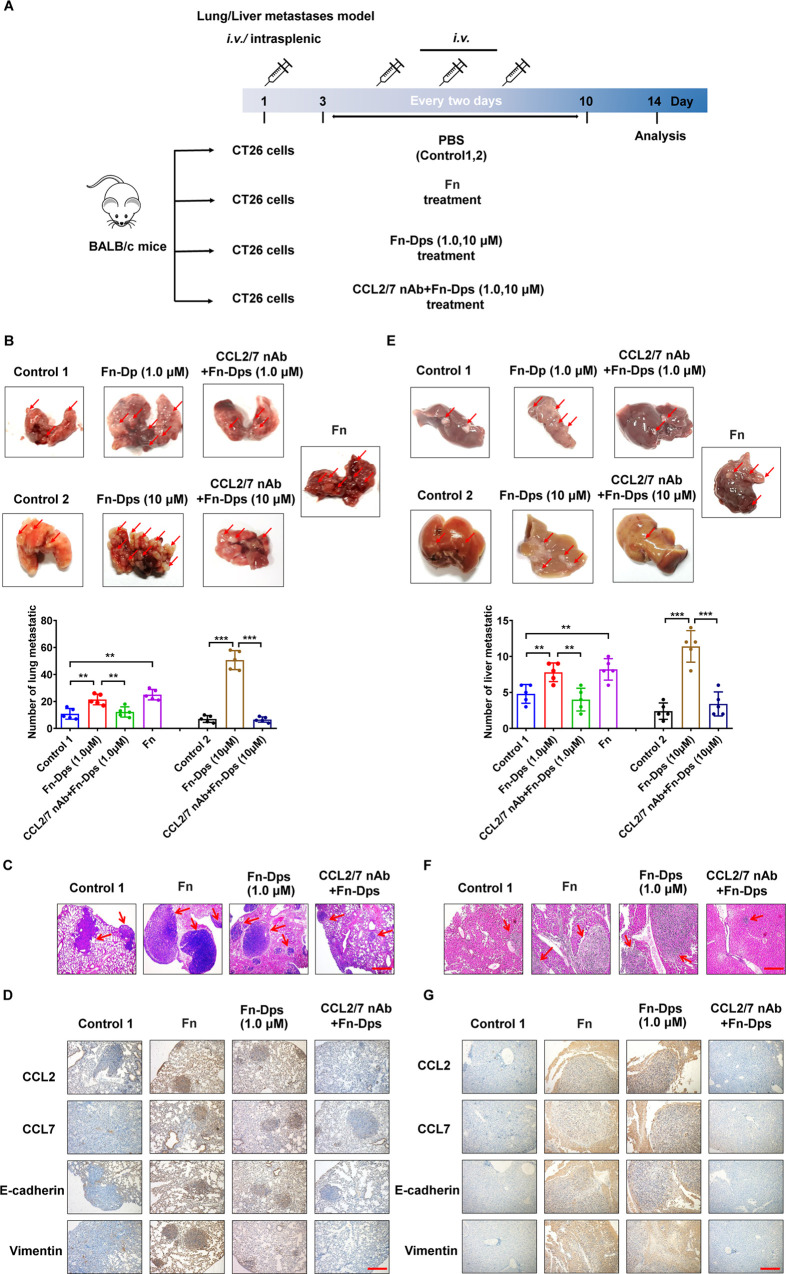
Fn-Dps promotes the migration of CRC cells *in vivo*. (A) A flow chart depicting the *in vivo* experimental design. (B) The representative photograph of the harvested lungs shows regions with multiple nodules and quantitative analysis (Below). (C) Histopathological examination of the lung tissue sections. (D) Immunohistochemical staining of CCL2, CCL7, E-cadherin, and Vimentin expression in paraffin-embedded lung tissues. (E) Representative photograph of the harvested liver showing regions with multiple nodules and quantitative analysis (Below). (F) Histopathological examination of the liver tissue sections. (G) Immunohistochemical staining of CCL2, CCL7, E-cadherin, and Vimentin expression in paraffin-embedded liver tissues. Scale bar  =  200 μm. Data are expressed as mean ± SD and compared by Student’s t test (B and E). *P<0.05, **P<0.01, ***P <0.001. n = 5 independent experiments.

To confirm the contribution of Fn-Dps to tumor metastasis *in vivo*. Fn-Dps activity was evaluated in two mouse colorectal tumor models. The scheme is presented in **Fig 8A**. In the lung metastasis model, the two Fn-Dps-treated groups presented a higher number of nodules than the control group (*P*<0.001), and more nodules were observed in high dose Fn-Dps-treated group (10 μM) than low dose group (1.0 μM) (*P*<0.05) **(Fig 8B)**. Metastatic dissemination induced by Fn-Dps was partly prevented by CCL2/7nAb (*P*<0.001) **(Fig 8B)**. Consistent results were also observed in H&E-stained lung sections, and the two Fn-Dps-treated groups exhibited a larger area and a higher number of cancer nests inside lung tissues than the control group **(Figs 8C and S16A)**. In addition, the expression of CCL2/7, E-cadherin, and Vimentin were increased, while E-cadherin was decreased in lung tissues of the Fn-Dps-treated group **(Figs 8D and S16B)**.

Similar results were obtained using the live metastasis model. The two Fn-Dps-treated groups had more tumor nodules distributed on the liver surface (*P*<0.01), while fewer metastases were observed in the CCL2/7 nAb + Fn-Dps group (*P*<0.05) **(Fig 8E)**. Liver sections by H&E staining showed that the cancer nest was obvious inside liver tissues of the Fn-Dps-treated groups, while less was observed in the CCL2/7 nAb+ Fn-Dps group **(Figs 8F and S16C)**. Moreover, the expression of CCL2/7, E-cadherin, and Vimentin were increased, while E-cadherin was decreased in liver tissues of the Fn-Dps-treated group **(Figs 8G and S16D)**. Collectively, these results suggest that Fn-Dps promotes tumor metastasis *in vivo* by CCL2/7-induced EMT.

## Discussion

Here, we demonstrated that Fn-Dps enhances the intracellular survival and replication of Fn in monocytes/macrophages. The ability to survive oxidative stress is a strong indicator in an intracellular bacterium of pathogenic potential. Dps protect DNA from oxidative stress by detoxifying H_2_O_2_ and have been implicated in intracellular survival and virulence such as *Campylobacter jejuni* and *Listeria monocytogenes* [24,25]. Moreover, the Dps from two obligate anaerobes *Porphyromonas gingivalis* (Pg) and *B*. *fragilis*, [26] were found to play an important role in the protection of cells from peroxide stress, and increase the survival of Pg inside endothelial cells.[27] As the most abundant cytoplasmic protein in the stationary phase, Dps is induced nearly 500-fold by H_2_O_2_ or oxygen exposure in Bf [28]. Similarly, Fn-Dps provides a protective role against intracellular H_2_O_2_ produced from macrophages. Together with these studies, our findings suggest that Fn-Dps is an important virulence protein to support the survival of Fn inside macrophages. It’s possible that macrophages carry live Fn into the tumor microenvironment, and transfer Fn to tumor cells to promote tumor progression.

We further demonstrated that ferritin Fn-Dps could induce erythrocyte damage *in vitro*. As Dps homologs, Fn-Dps can store iron and play a key role in iron homeostasis. Intriguingly, we also identified that Fn released hemin receptor (Q8RG20) and hemolysin activator protein (Q8RGK1) under conditions of nutritional limitation **(S2 Table)**. The ability to acquire iron during infection is an essential attribute of many bacterial pathogens. To successfully mediate infection, some pathogens have developed a heme uptake system and are capable of scavenging heme iron during infection [29]. Similar to these pathogens, our findings hinted that erythrocytes were damaged and heme was hijacked by hemolysin activator protein and hemin receptor, and then heme iron was scavenged by Fn-Dps during Fn infection.

Fn-Dps shared less than 30% identity with Hp-Dps protein. Hp is etiologically linked to gastritis and gastric cancer. The clinical outcome of Hp infection is determined by a complex interaction of environmental influences, host factors, and microbial virulence factors [30]. Hp-Dps was initially defined as neutrophil-activating protein (Hp-Nap) that activates neutrophils and monocytes [31]. Notably, Hp-Nap is a fundamental virulence factor that exhibits chemotactic activity in human leukocytes [32], and promotes the adhesion of neutrophils to endothelial cells [33]. Our study showed that Fn-Dps plays a similar chemotactic role in macrophages.

Tumor cell migration and metastasis share many similarities with leukocyte trafficking, which is mainly mediated by chemokines and their receptors. Macrophages are the most abundant stromal cells in multiple malignancies. The crosstalk between tumor and tumor-associated macrophages (TAM) can promote tumor malignancy through crosstalk-derived chemokines. Several TAM-secreted chemokines, including CCL2/7, have been identified to be associated with tumor progression [34–37]. Our study reveals that both Fn-Dps-stimulated and Fn-infected macrophages secrete the chemokine CCL2/7. Both immune cell-driven CCL2/7 can enhance tumor metastasis by promoting EMT or recruiting macrophages in CRC [38,39]. CCL7 was found to interplay with CC chemokine receptor 3 (CCR3), resulting in enhanced invasion and migration [40]. Together with our previous study showing that Fn can multiply in macrophages, our results demonstrated that Fn-infected macrophages could promote metastasis by Fn-Dps-regulated EMT process of CRC cells in the tumor microenvironment.

Moreover, the development of vaccines is an ideal strategy to eliminate infections. However, vaccination of mice with inactivated Fn fails to prevent experimental periodontitis [41]. Subunit vaccines FomA and AhpC can partly reduce the Fn load in the mouse intestinal tract but cannot eliminate or eradicate Fn infection completely [23,42]. Curiously, in our study, systemic immunization with the Fn-Dps /Alum group, which elicited extremely high serum IgG titers against Fn-Dps, resulted in an increased Fn load in the cecum. The results indicated that overly high anti-Fn-Dps IgG levels might drive Fn bacteria to accumulate in the cecum.

Our investigation consistently showed a higher titer of anti-Fn-Dps antibodies present in the serum of CRC patients than that of healthy subjects. Similarly, elevated anti-Hp-Nap antibodies were observed in patients with gastritis and gastric cancer [43]. Hp-Nap has been identified as a potential diagnostic biomarker and vaccine candidate for gastric cancer [44,45]. However, a recent clinical trial showed that three recombinant antigens, including Hp-Nap, did not confer additional protection against Hp infection compared with placebo in healthy subjects, despite increased systemic humoral responses to these antigens [46]. Together with our study, these investigations suggested that the immune strategy to eliminate tumor-associated bacteria faces significant challenges. Our study showed that high anti-Fn-Dps SIgA levels conferred protection against Fn load, so developing a mucosal delivery system for Fn-Dps vaccines might effectively reduce the Fn load in the gut.

**Fig 9:**
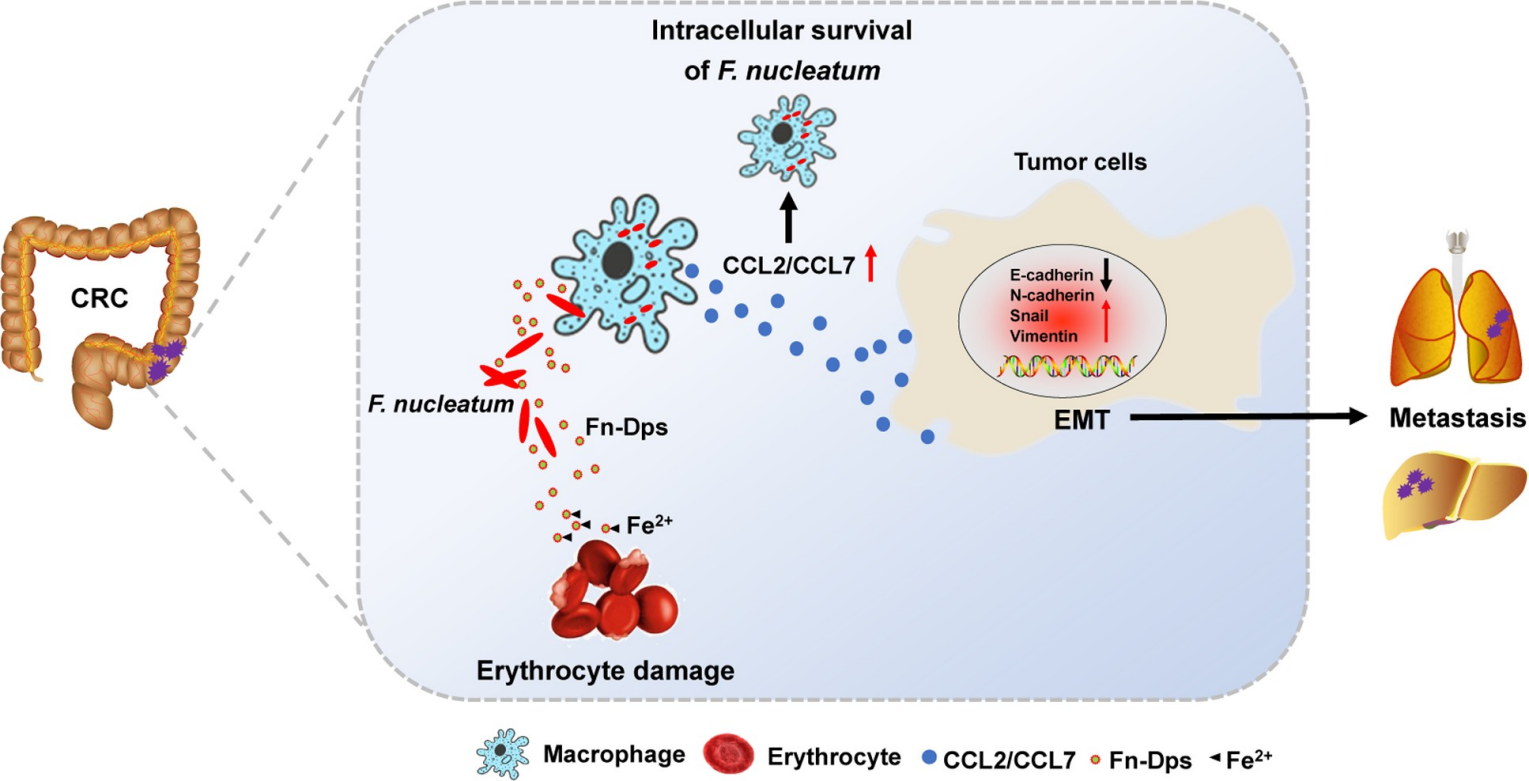
A schematic model of Fn-Dps functions in tumor metastasis. The illustration was drawn using ScienceSlides Software and Microsoft Office PowerPoint Software.

In summary, we identified Fn-Dps as an important virulence factor in the pathogenesis of Fn infection. Fn-Dps contributes to its survival in macrophages and induces erythrocyte damage. Moreover, Fn-Dps promotes the migration of CRC cells *via* CCL2/7-induced EMT **(Fig 9)**. We also found elevated anti-Fn-Dps antibodies in CRC patients and demonstrated mucosal immunization with Fn-Dps conferred protection against Fn. Our results further support that Fn is an oncogenic microorganism based on the important virulence factor Fn-Dps.

## Supporting information

S1 Fig Phylogenetic conservation analysis by the NCBI conserved domain search tool. (PDF)

S2 Fig SDS-PAGE of Fn-Dps-induced expression with 0.8 mM IPTG. (PDF)

S3 Fig Erythrocytes were stained with Wright’s staining solution in mouse erythrocytes after 8 h posttreatment with Fn (MOI, 10:1). (PDF)

S4 Fig The cytotoxic effect of Fn-Dps (1.0 μM) on J774A.1 cells were evaluated after treatment with Fn-Dps for indicated time using the CCK-8 assay. Data are expressed as mean ± SD and compared by One-way ANOVA.
(PDF)

S5 Fig Morphological changes of J774A.1 cells stimulated by Fn-Dps (1.0 μM) for 24 h. Scale bar  =  400 μm.
(PDF)

S6 Fig Heatmap of differentially expressed genes identified through RNA-seq analysis (log2 fold change>2, and adjusted *P*<0.05). Heatmap showing RNA-seq data from RAW264.7 cells after Fn-Dps (A) or Fn (B) treatment.
(PDF)

S7 Fig Gene Ontology (GO) enrichment analysis of the Up-regulated or down-regulated genes between the control and Fn-Dps treated RAW264.7 cells. (PDF)

S8 Fig KEGG pathway analysis of the Up-regulated or down-regulated genes between the control and Fn-Dps treated RAW264.7 cells. (PDF)

S9 Fig RAW264.7 cells stimulated by Fn or *B*. *fragilis* (MOI = 10:1). CCL7, STFA3, CSF3, CCL2 mRNA levels were validated by RT–qPCR. Data are expressed as mean ± SD and compared by Student’s t test. *P<0.05, **P<0.01, ***P <0.001. n = 3 independent experiments.
(PDF)

S10 Fig Gentamycin susceptibility testing for Fn by E-test. (PDF)

S11 Fig (A) Gentamycin susceptibility testing for *B*. *fragilis* by E-test. (B) RAW264.7 cells were infected with *B*. *fragilis* at an MOI of 10 for 12h, followed by gentamicin treatment to eliminate extracellular bacteria. The survival of intracellular *B*. *fragilis* in RAW264.7 cells was examined with colony-forming unit (CFU) assay.
(PDF)

S12 Fig Fn-Dps promotes the migration of RKO cells. (A) Schematic diagram of the RKO cells migration model: (a) RKO cells were seeded in the top well, and macrophages derived from THP-1 cells (M-THP-1) were added to the lower wells treated with PBS (control), (b) or treated with Fn-Dps, (c) or treated with CCL2, (d) or treated with CCL7, (e) or treated with CCL2+CCL7, (f) or treated with CCL2/7 nAb+Fn-Dps. Representative images of the assay (right). (B) The migration of RKO cells was assessed using Transwell migration assays. Representative images of the assay (right). (C) Analysis of RKO cells migration by scratch assays. RKO cells were treated with supernatant of M-Thp-1cells alone (control), with CCL2, with CCL7, with CCL2+CCL7, or with supernatant of M-Thp-1 cells treated with CCL2/7nAb+Fn-Dps. Scratch area was recorded after treatment for 48 h. Representative images of the assay (right). (D) RKO cells were treated with supernatant of M-Thp-1 cells alone (control), with CCL2, with CCL7, with CCL2+CCL7, or with supernatant of M-Thp-1 cells treated with CCL2/7nAb+Fn-Dps for 48 h. The expression of E-cadherin, N-cadherin, Snail and Vimentin was measured by Western blot analysis. Scale bar  =  200 μm. Data are expressed as mean ± SD and compared by Student’s t test (B and C). *P<0.05, **P<0.01, ***P <0.001. n = 3 independent experiments.
(PDF)

S13 Fig The predicted antigenic peptides (BPAP) system was used to predict the antigenic plot for the Fn-Dps protein. (PDF)

S14 Fig Serum antibody responses and the induction of protection against Fn by immunization with Fn-Dps. (A) Schematic of immunization experiments in mice. Mice were immunized by subcutaneous injection (*sc*.) with PBS, adjuvant aluminum hydroxide (Alum), Fn-Dps or Fn-Dps combined with adjuvant. (B) The anti-Fn-Dps IgG titer; (C) The anti-Fn-Dps IgA titer; (D) The anti-Fn-Dps SIgA titer. One week after the final vaccination, Fn-Dps antibody titers in sera/intestinal mucus were determined using ELISA. Colonization of Fn in the colon (E) or cecum (F) of mice perfused with subcutaneous injection with PBS, Fn-Dps or Fn-Dps combined with alum. Colonization quantified using qPCR assay. The rate of protection was calculated using the following formula: protection rate (%) = (expression of Fn-DNA in the control group-expression of Fn-DNA in the immunized group)/expression of Fn-DNA in the control group × 100%. ^^represents none detected. Data are expressed as mean ± SD and compared by Student’s t test (B,C,D,E and F). *P<0.05, **P<0.01, ***P <0.001. n = 5 independent experiments.
(PDF)

S15 Fig Serum antibody responses by immunization with Fn-Dps. Mice were immunized by subcutaneous injection (*s*.*c*.) or by intragastric administration (*i*.*g*.) with PBS, adjuvant aluminum hydroxide (Alum) or cholera toxin B subunit (CTB), Fn-Dps or Fn-Dps combined with adjuvant. (A) The anti-Fn-Dps IgG titre; (B) The anti-Fn-Dps IgA titre; (C) The anti-Fn-Dps SIgA titre was detected by ELISA at the indicated times. Data are expressed as mean ± SD.
(PDF)

S16 Fig Fn-Dps promotes the migration of CRC cells *in vivo*. (A) Histopathological examination of the lung tissue sections. (B) Immunohistochemical staining of CCL2, CCL7, E-cadherin and Vimentin expression in paraffin-embedded lung tissues. (C) Histopathological examination of the liver tissue sections. (D) Immunohistochemical staining of CCL2, CCL7, E-cadherin and Vimentin expression in paraffin-embedded liver tissues. Scale bar  =  200 μm.
(PDF)

S17 Fig Full-length gels/blots of data represented in Fig 5D and S12D Fig. (PDF)

S18 Fig Full-length gels/blots of data represented in Fig 7A and7B. (PDF)

S1 Table Sequence of primer. (PDF)

S2 Table Mass spectrometry (MS)-identified soluble protein of the culture supernatant of Fn under starved conditions. (PDF)

S3 Table Mass spectrometry (MS)-identified soluble protein of the culture supernatant of Fn under BHI. (PDF)

S4 Table Homology analysis of Fn-Dps with *Fusobacterium*. (PDF)

S5 Table Homology analysis of Fn-Dps with other bacteria. (PDF)

S6 Table Prediction of antigenic determinants for the Fn-Dps protein among Fn and other related bacteria. (PDF)

S7 Table Relationship between the OD value of IgG/IgA antibodies against Fn-Dps and the clinicopathological variables in 123 patients with CRC. (PDF)
