# Supplementary figures and images for: Fn-Dps, a novel virulence factor of Fusobacterium nucleatum, disrupts erythrocytes and promotes metastasis in colorectal cancer
Source: PLoS Pathog. 2023 Jan 24;19(1):e1011096. doi: 10.1371/journal.ppat.1011096 (PMC9873182; doi:10.1371/journal.ppat.1011096)

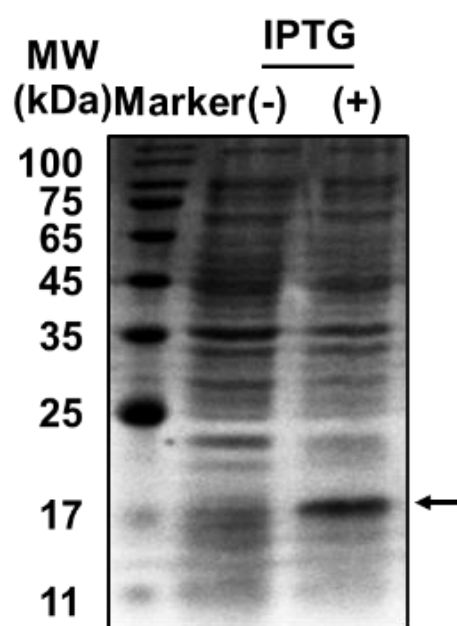

**S2 Fig.** SDS-PAGE of Fn-Dps-induced expression with 0.8 mM IPTG.

Supplement: S2 Fig — (PDF) [file ppat.1011096.s002.pdf]

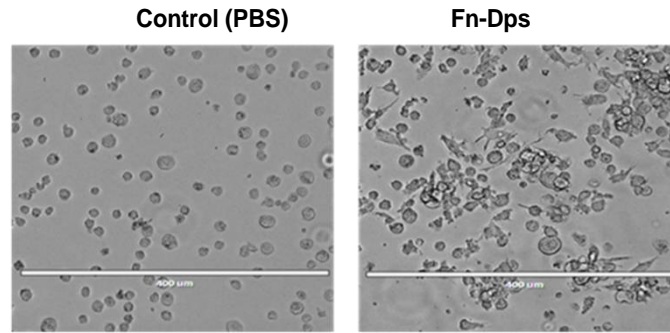

**S5 Fig.** Morphological changes of J774A.1 cells stimulated by Fn-Dps (1.0  $\mu$ M) for 24 h. Scale bar = 400  $\mu$ m.

Supplement: S5 Fig — Scale bar = 400 μm. (PDF) [file ppat.1011096.s005.pdf]

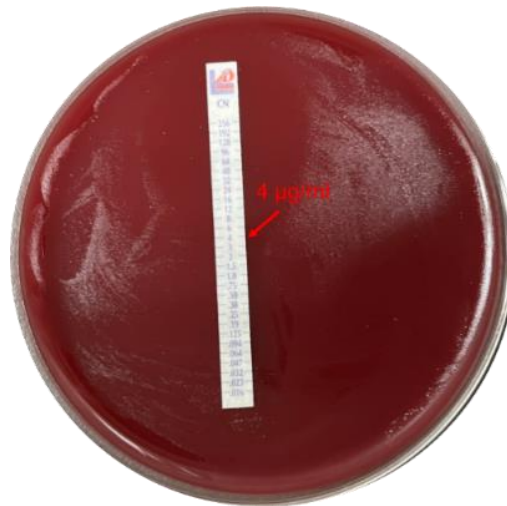

**S10 Fig.** Gentamycin susceptibility testing for Fn by E-test.

Supplement: S10 Fig — (PDF) [file ppat.1011096.s010.pdf]

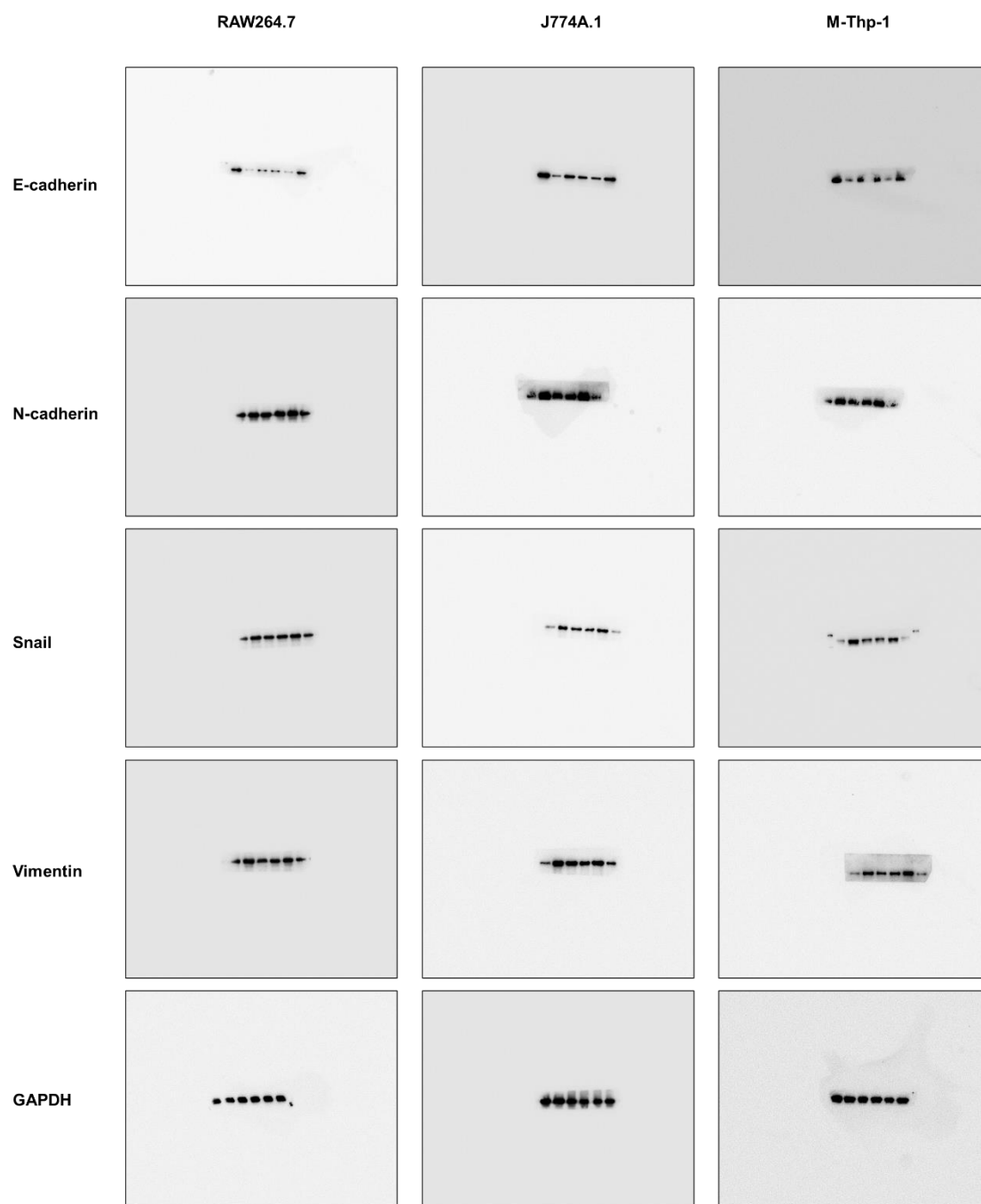

**S17 Fig.** Full-length gels/blots of data represented in Fig 5D and S12D Fig.

Supplement: S17 Fig — (PDF) [file ppat.1011096.s017.pdf]

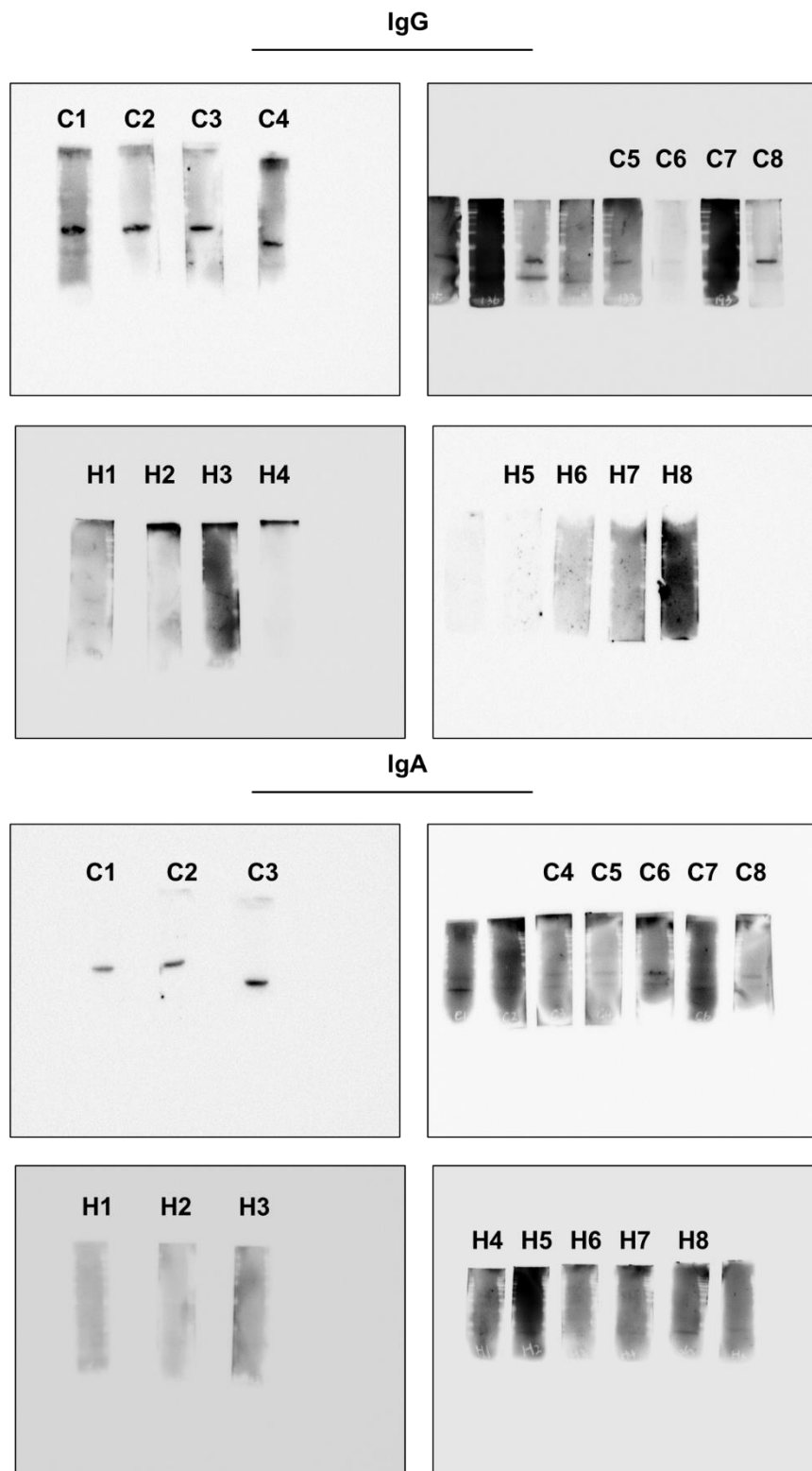

**S18 Fig.** Full-length gels/blots of data represented in Fig 7A and 7B.

Supplement: S18 Fig — (PDF) [file ppat.1011096.s018.pdf]
