# Supplementary material for: Fn-Dps, a novel virulence factor of Fusobacterium nucleatum, disrupts erythrocytes and promotes metastasis in colorectal cancer
Source: PLoS Pathog. 2023 Jan 24;19(1):e1011096. doi: 10.1371/journal.ppat.1011096 (PMC9873182; doi:10.1371/journal.ppat.1011096)
Supplement: S3 Fig — (PDF) [file ppat.1011096.s003.pdf]

Fn

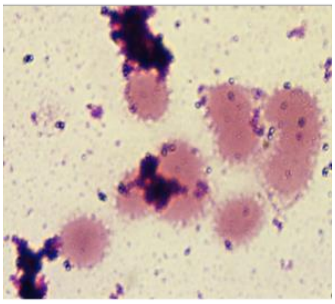

S3 Fig. Erythrocytes were stained with Wright's staining solution in mouse erythrocytes after 8 h posttreatment with Fn (MOI, 10:1).
