# Supplementary material for: Fn-Dps, a novel virulence factor of Fusobacterium nucleatum, disrupts erythrocytes and promotes metastasis in colorectal cancer
Source: PLoS Pathog. 2023 Jan 24;19(1):e1011096. doi: 10.1371/journal.ppat.1011096 (PMC9873182; doi:10.1371/journal.ppat.1011096)
Supplement: S4 Fig — Data are expressed as mean ± SD and compared by One-way ANOVA. (PDF) [file ppat.1011096.s004.pdf]

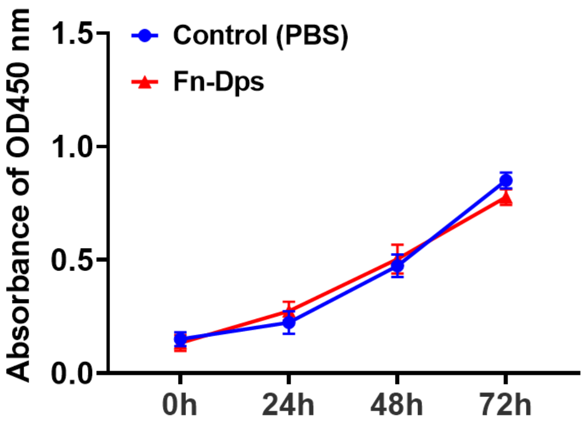

S4 Fig. The cytotoxic effect of Fn-Dps (1.0 μM) on J774A.1 cells were evaluated after treatment with Fn-Dps for indicated time using the CCK-8 assay. Data are expressed as mean ± SD and compared by One-way ANOVA.
