# Supplementary material for: Fn-Dps, a novel virulence factor of Fusobacterium nucleatum, disrupts erythrocytes and promotes metastasis in colorectal cancer
Source: PLoS Pathog. 2023 Jan 24;19(1):e1011096. doi: 10.1371/journal.ppat.1011096 (PMC9873182; doi:10.1371/journal.ppat.1011096)
Supplement: S6 Fig — Heatmap showing RNA-seq data from RAW264.7 cells after Fn-Dps (A) or Fn (B) treatment. (PDF) [file ppat.1011096.s006.pdf]

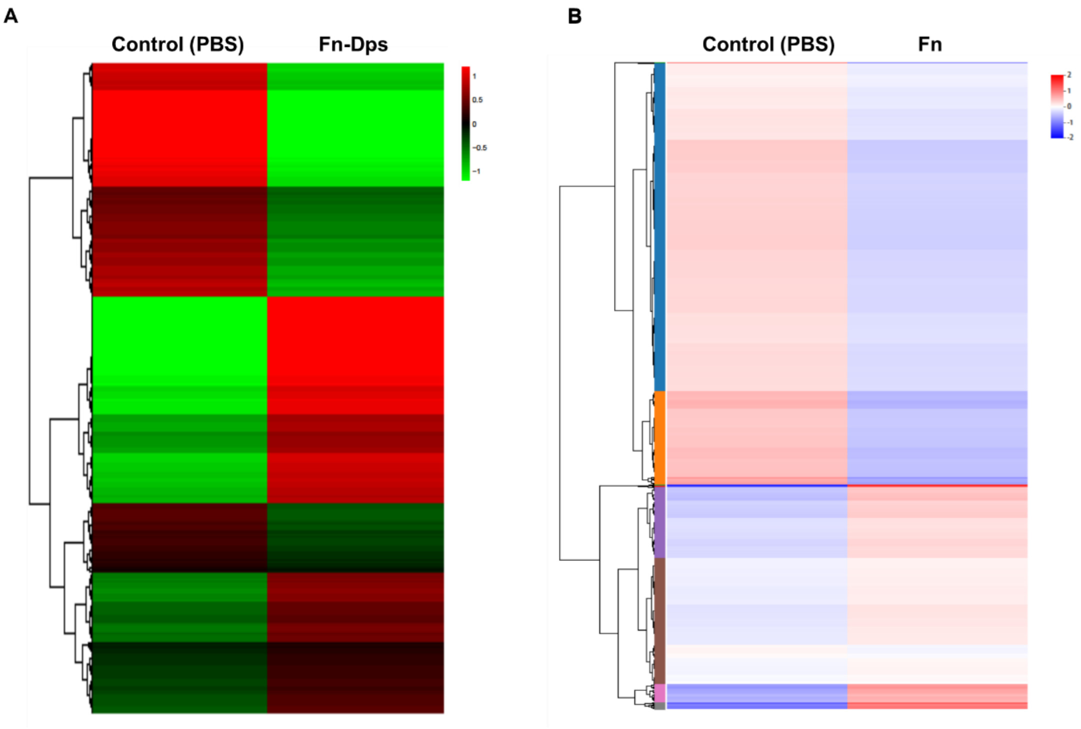

S6 Fig. Heatmap of differentially expressed genes identified through RNA-seq analysis (log2 fold change>2, and adjusted *P*<0.05). Heatmap showing RNA-seq data from RAW264.7 cells after Fn-Dps **(A)** or Fn **(B)** treatment.
