# Supplementary material for: Fn-Dps, a novel virulence factor of Fusobacterium nucleatum, disrupts erythrocytes and promotes metastasis in colorectal cancer
Source: PLoS Pathog. 2023 Jan 24;19(1):e1011096. doi: 10.1371/journal.ppat.1011096 (PMC9873182; doi:10.1371/journal.ppat.1011096)
Supplement: S7 Fig — (PDF) [file ppat.1011096.s007.pdf]

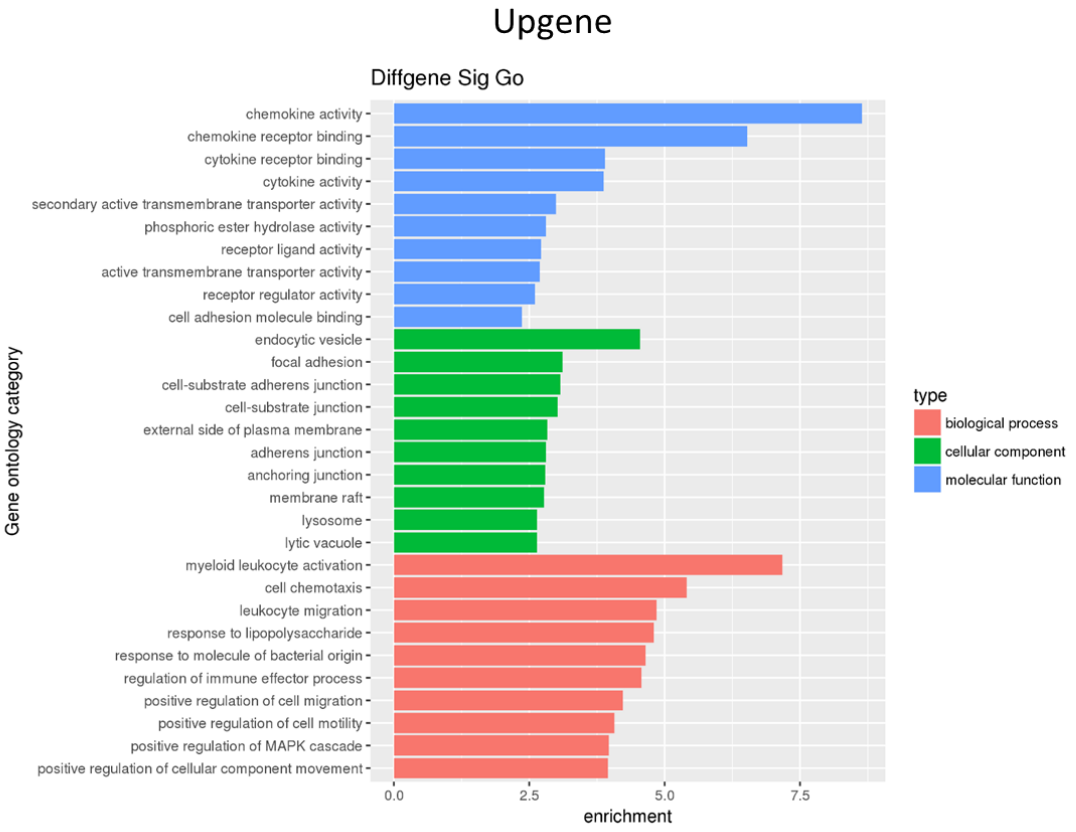

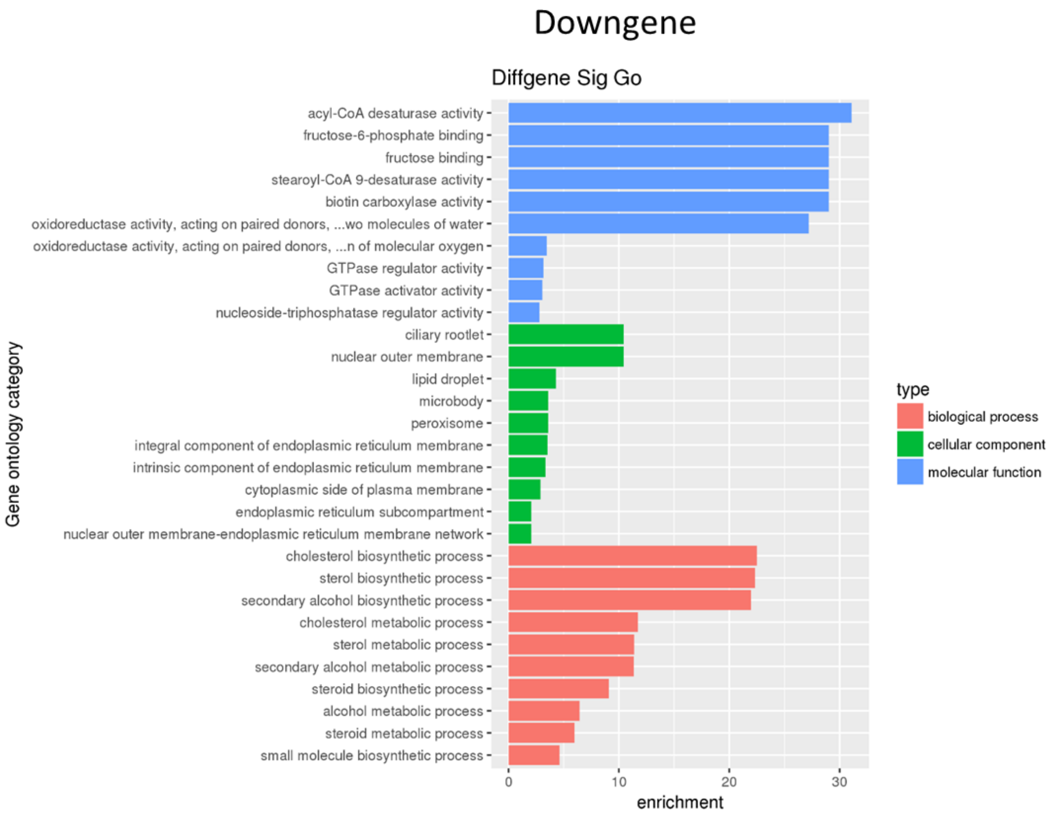

S7 Fig. Gene Ontology (GO) enrichment analysis of the up-regulated or down-regulated genes between the control and Fn-Dps treated RAW264.7 cells.
