# Supplementary material for: Fn-Dps, a novel virulence factor of Fusobacterium nucleatum, disrupts erythrocytes and promotes metastasis in colorectal cancer
Source: PLoS Pathog. 2023 Jan 24;19(1):e1011096. doi: 10.1371/journal.ppat.1011096 (PMC9873182; doi:10.1371/journal.ppat.1011096)
Supplement: S9 Fig — CCL7, STFA3, CSF3, CCL2 mRNA levels were validated by RT–qPCR. Data are expressed as mean ± SD and compared by Student’s t test. *P<0.05, **P<0.01, ***P <0.001. n = 3 independent experiments. (PDF) [file ppat.1011096.s009.pdf]

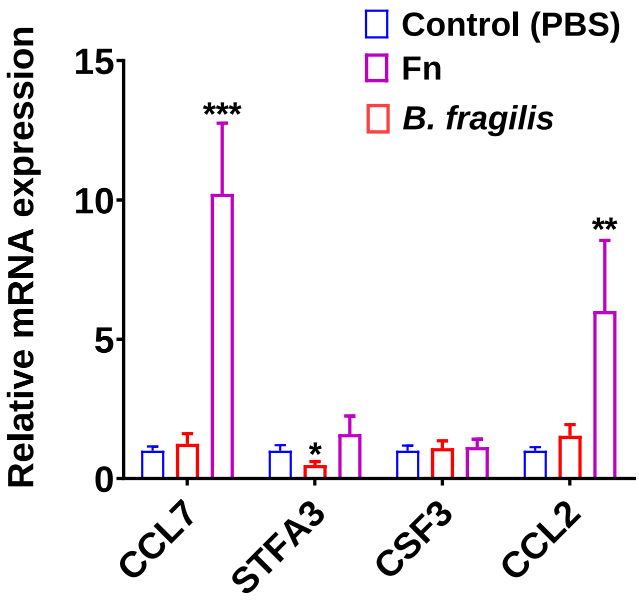

S9 Fig. RAW264.7 cells stimulated by Fn or *B. fragilis* (MOI=10:1). CCL7, STFA3, CSF3, CCL2 mRNA levels were validated by RT–qPCR. Data are expressed as mean ± SD and compared by Student's t test. **P*<0.05, ***P*<0.01, ****P* <0.001. n = 3 independent experiments.
