# Supplementary material for: Fn-Dps, a novel virulence factor of Fusobacterium nucleatum, disrupts erythrocytes and promotes metastasis in colorectal cancer
Source: PLoS Pathog. 2023 Jan 24;19(1):e1011096. doi: 10.1371/journal.ppat.1011096 (PMC9873182; doi:10.1371/journal.ppat.1011096)
Supplement: S11 Fig — (A) Gentamycin susceptibility testing for B. fragilis by E-test. (B) RAW264.7 cells were infected with B. fragilis at an MOI of 10 for 12h, followed by gentamicin treatment to eliminate extracellular bacteria. The survival of intracellular B. fragilis in RAW264.7 cells was examined with colony-forming unit (CFU) assay. (PDF) [file ppat.1011096.s011.pdf]

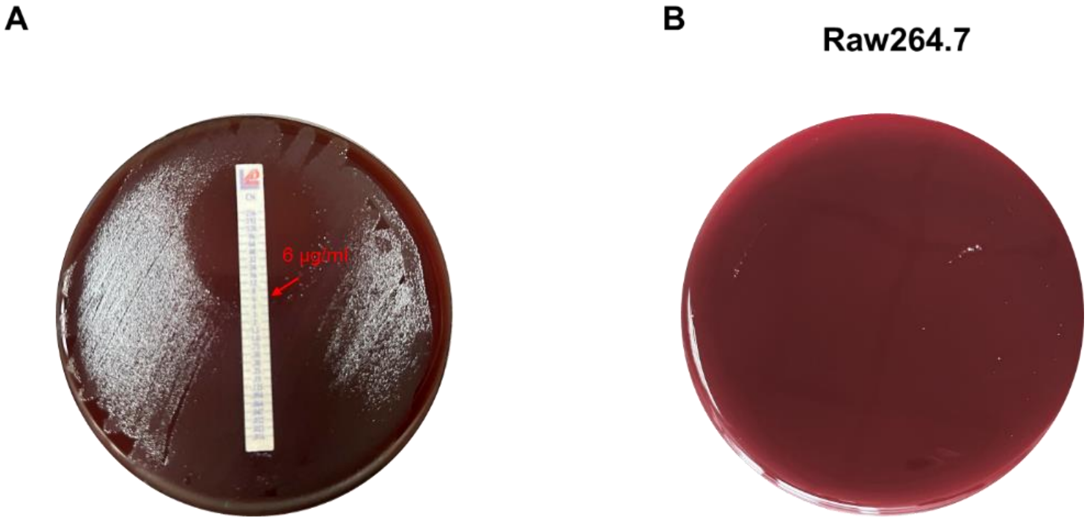

S11 Fig. (A) Gentamycin susceptibility testing for *B. fragilis* by E-test**. (B)** RAW264.7 cells were infected with *B. fragilis* at an MOI of 10 for 12h, followed by gentamicin treatment to eliminate extracellular bacteria. The survival of intracellular *B. fragilis* in RAW264.7 cells was examined with colony-forming unit (CFU) assay.
