# Supplementary material for: Fn-Dps, a novel virulence factor of Fusobacterium nucleatum, disrupts erythrocytes and promotes metastasis in colorectal cancer
Source: PLoS Pathog. 2023 Jan 24;19(1):e1011096. doi: 10.1371/journal.ppat.1011096 (PMC9873182; doi:10.1371/journal.ppat.1011096)
Supplement: S12 Fig — (A) Schematic diagram of the RKO cells migration model: (a) RKO cells were seeded in the top well, and macrophages derived from THP-1 cells (M-THP-1) were added to the lower wells treated with PBS (control), (b) or treated with Fn-Dps, (c) or treated with CCL2, (d) or treated with CCL7, (e) or treated with CCL2+CCL7, (f) or treated with CCL2/7 nAb+Fn-Dps. Representative images of the assay (right). (B) The migration of RKO cells was assessed using Transwell migration assays. Representative images of the assay (right). (C) Analysis of RKO cells migration by scratch assays. RKO cells were treated with supernatant of M-Thp-1cells alone (control), with CCL2, with CCL7, with CCL2+CCL7, or with supernatant of M-Thp-1 cells treated with CCL2/7nAb+Fn-Dps. Scratch area was recorded after treatment for 48 h. Representative images of the assay (right). (D) RKO cells were treated with supernatant of M-Thp-1 cells alone (control), with CCL2, with CCL7, with CCL2+CCL7, or with supernatant of M-Thp-1 cells treated with CCL2/7nAb+Fn-Dps for 48 h. The expression of E-cadherin, N-cadherin, Snail and Vimentin was measured by Western blot analysis. Scale bar = 200 μm. Data are expressed as mean ± SD and compared by Student’s t test (B and C). *P<0.05, **P<0.01, ***P <0.001. n = 3 independent experiments. (PDF) [file ppat.1011096.s012.pdf]

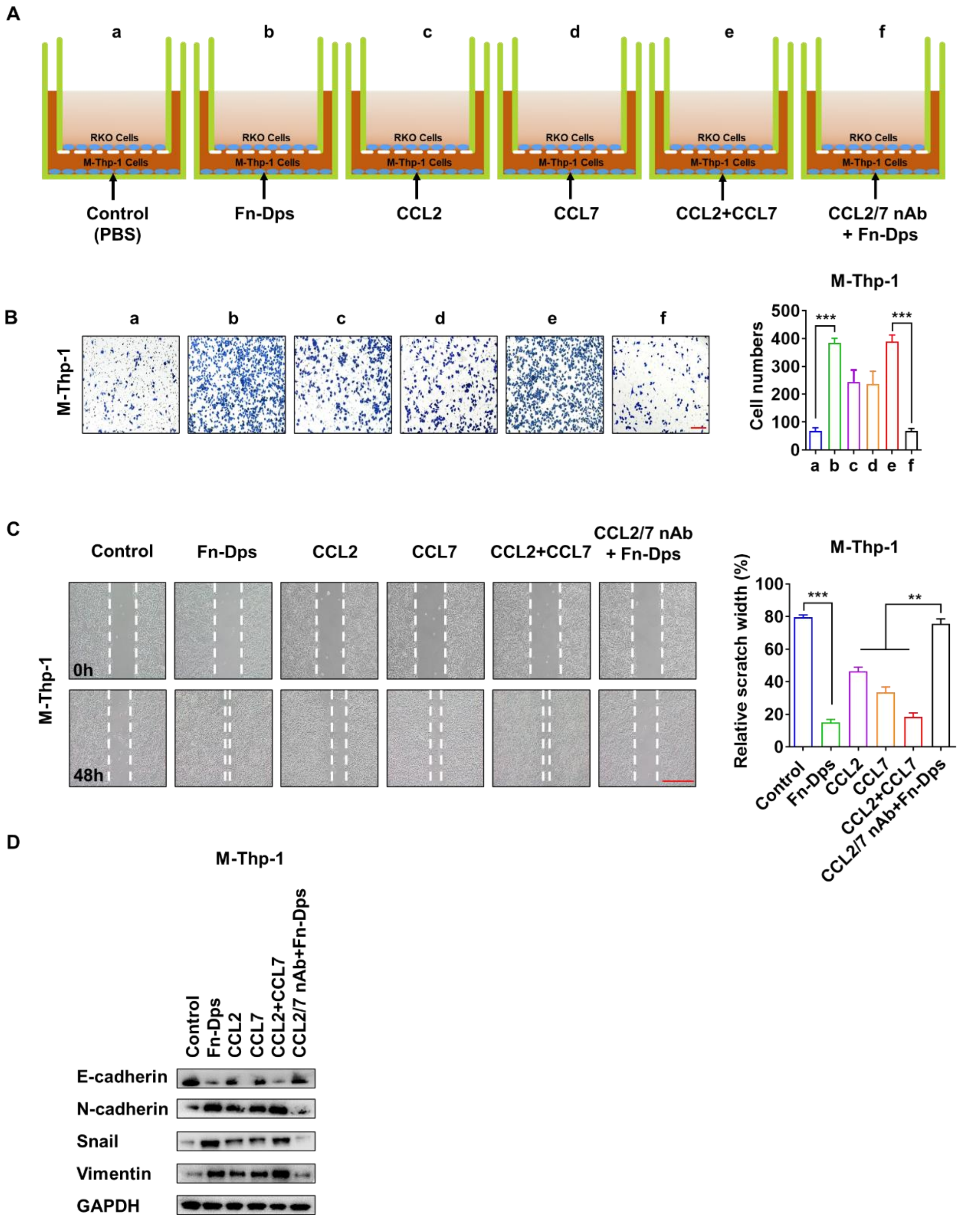

S12 Fig. Fn-Dps promotes the migration of RKO cells. (A) Schematic diagram of the RKO cells migration model**:** (a) RKO cells were seeded in the top well, and macrophages derived from THP-1 cells (M-THP-1) were added to the lower wells treated with PBS (control), (b) or treated with Fn-Dps, (c) or treated with CCL2, (d) or treated with CCL7, (e) or treated with CCL2+CCL7, (f) or treated with CCL2/7 nAb+Fn-Dps. Representative images of the assay (right). **(B)** The migration of RKO cells was assessed using Transwell migration assays. Representative images of the

assay (right). **(C)** Analysis of RKO cells migration by scratch assays. RKO cells were treated with supernatant of M-Thp-1cells alone (control), with CCL2, with CCL7, with CCL2+CCL7, or with supernatant of M-Thp-1 cells treated with CCL2/7nAb+Fn-Dps. Scratch area was recorded after treatment for 48 h. Representative images of the assay (right). **(D)** RKO cells were treated with supernatant of M-Thp-1 cells alone (control), with CCL2, with CCL7, with CCL2+CCL7, or with supernatant of M-Thp-1 cells treated with CCL2/7nAb+Fn-Dps for 48 h. The expression of E-cadherin, N-cadherin, Snail and Vimentin was measured by Western blot analysis. Scale bar = 200 μm. Data are expressed as mean ± SD and compared by Student's t test (B and C). *P<0.05, **P<0.01, ***P <0.001. n = 3 independent experiments.
