# Supplementary material for: Fn-Dps, a novel virulence factor of Fusobacterium nucleatum, disrupts erythrocytes and promotes metastasis in colorectal cancer
Source: PLoS Pathog. 2023 Jan 24;19(1):e1011096. doi: 10.1371/journal.ppat.1011096 (PMC9873182; doi:10.1371/journal.ppat.1011096)
Supplement: S13 Fig — (PDF) [file ppat.1011096.s013.pdf]

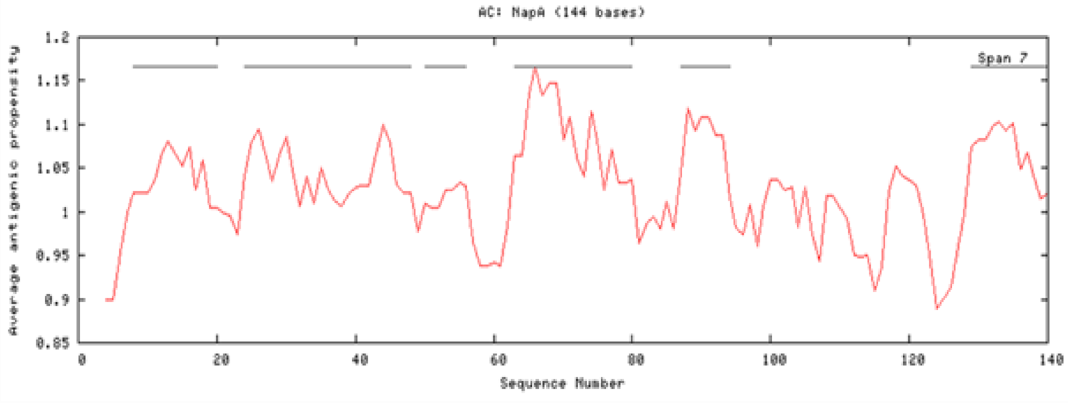

MKNKENLNKY LSNLGILITK THNLHWNV VGARFKAI
HEYTESLYDY YFEKFDEVAE AFKMKGEFPL VKVADYLKHA
TVKELEAKDF TIPEVVTSIKE DIELMLADAR KIREVANEED
DFLVANMMED QIEYFVKQLW FISAMAK

S13 Fig. The predicted antigenic peptides (BPAP) system was used to predict the antigenic plot for the Fn-Dps protein.
