# Supplementary material for: Fn-Dps, a novel virulence factor of Fusobacterium nucleatum, disrupts erythrocytes and promotes metastasis in colorectal cancer
Source: PLoS Pathog. 2023 Jan 24;19(1):e1011096. doi: 10.1371/journal.ppat.1011096 (PMC9873182; doi:10.1371/journal.ppat.1011096)
Supplement: S14 Fig — (A) Schematic of immunization experiments in mice. Mice were immunized by subcutaneous injection (sc.) with PBS, adjuvant aluminum hydroxide (Alum), Fn-Dps or Fn-Dps combined with adjuvant. (B) The anti-Fn-Dps IgG titer; (C) The anti-Fn-Dps IgA titer; (D) The anti-Fn-Dps SIgA titer. One week after the final vaccination, Fn-Dps antibody titers in sera/intestinal mucus were determined using ELISA. Colonization of Fn in the colon (E) or cecum (F) of mice perfused with subcutaneous injection with PBS, Fn-Dps or Fn-Dps combined with alum. Colonization quantified using qPCR assay. The rate of protection was calculated using the following formula: protection rate (%) = (expression of Fn-DNA in the control group-expression of Fn-DNA in the immunized group)/expression of Fn-DNA in the control group × 100%. ^^represents none detected. Data are expressed as mean ± SD and compared by Student’s t test (B,C,D,E and F). *P<0.05, **P<0.01, ***P <0.001. n = 5 independent experiments. (PDF) [file ppat.1011096.s014.pdf]

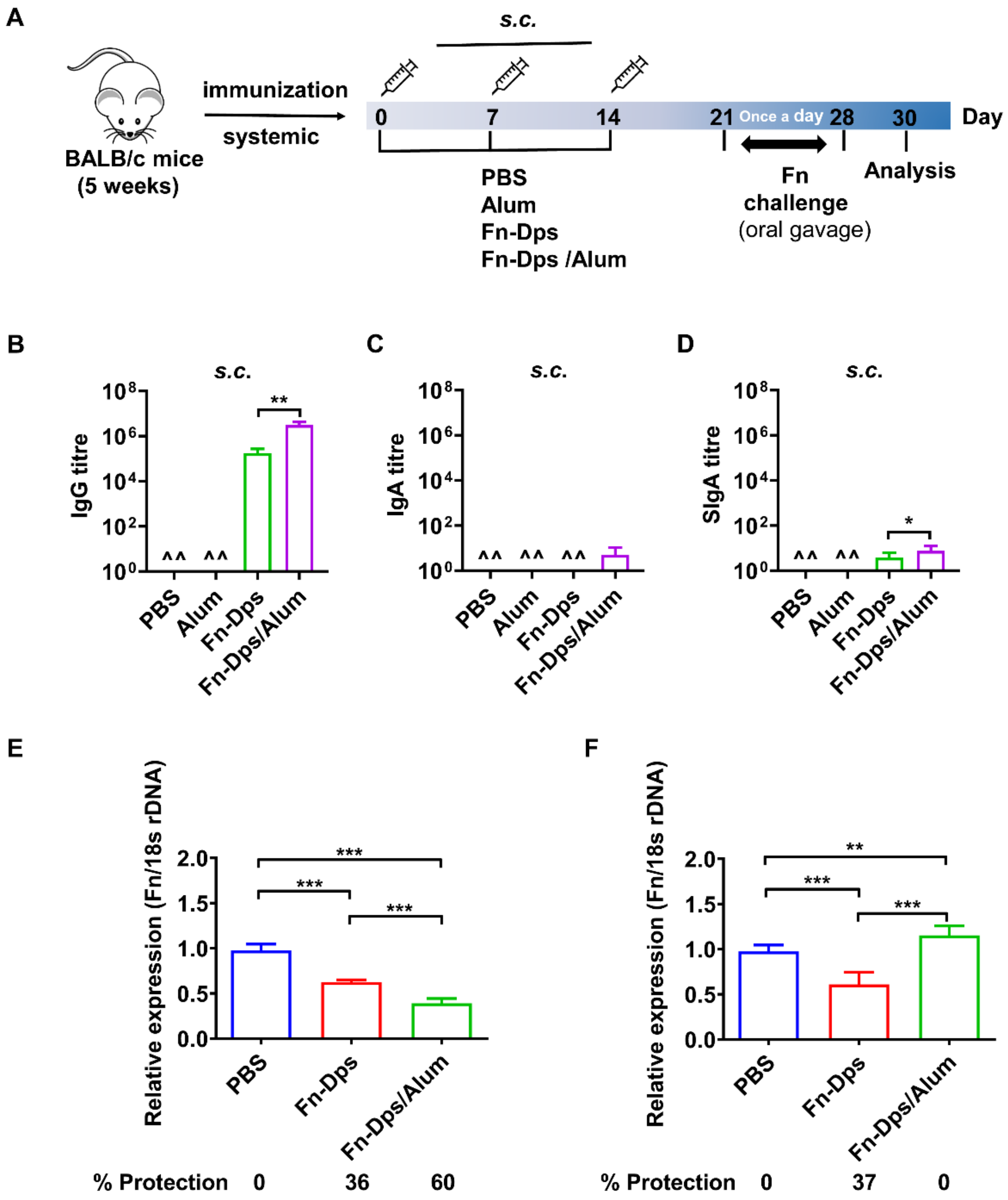

S14 Fig. Serum antibody responses and the induction of protection against Fn by immunization with Fn-Dps. **(A)** Schematic of immunization experiments in mice. Mice were immunized by subcutaneous injection (*sc.*) with PBS, adjuvant aluminum hydroxide (Alum), Fn-Dps or Fn-Dps combined with adjuvant. **(B)**The anti-Fn-Dps IgG titer; **(C)**The anti-Fn-Dps IgA titer; **(D)**The anti-Fn-Dps SIgA titer. One week after the final vaccination, Fn-Dps antibody titers in sera/intestinal mucus were determined using ELISA. Colonization of Fn in the colon **(E)** or cecum **(F)** of mice perfused with subcutaneous injection with PBS, Fn-Dps or Fn-Dps combined with

alum. Colonization quantified using qPCR assay. The rate of protection was calculated using the following formula: protection rate (%) = (expression of Fn-DNA in the control group-expression of Fn-DNA in the immunized group)/expression of Fn-DNA in the control group × 100%. ^^represents none detected. Data are expressed as mean ± SD and compared by Student's t test (B,C,D,E and F). *P<0.05, **P<0.01, ***P <0.001. n = 5 independent experiments.
