# Supplementary material for: Fn-Dps, a novel virulence factor of Fusobacterium nucleatum, disrupts erythrocytes and promotes metastasis in colorectal cancer
Source: PLoS Pathog. 2023 Jan 24;19(1):e1011096. doi: 10.1371/journal.ppat.1011096 (PMC9873182; doi:10.1371/journal.ppat.1011096)
Supplement: S15 Fig — Mice were immunized by subcutaneous injection (s.c.) or by intragastric administration (i.g.) with PBS, adjuvant aluminum hydroxide (Alum) or cholera toxin B subunit (CTB), Fn-Dps or Fn-Dps combined with adjuvant. (A) The anti-Fn-Dps IgG titre; (B) The anti-Fn-Dps IgA titre; (C) The anti-Fn-Dps SIgA titre was detected by ELISA at the indicated times. Data are expressed as mean ± SD. (PDF) [file ppat.1011096.s015.pdf]

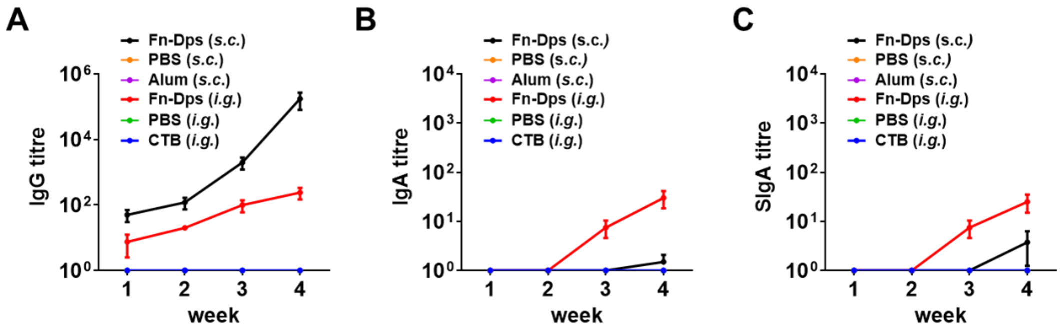

S15 Fig. Serum antibody responses by immunization with Fn-Dps. Mice were immunized by subcutaneous injection (*s.c.*) or by intragastric administration (*i.g.*) with PBS, adjuvant aluminum hydroxide (Alum) or cholera toxin B subunit (CTB), Fn-Dps or Fn-Dps combined with adjuvant. **(A)** The anti-Fn-Dps IgG titre; **(B)**The anti-Fn-Dps IgA titre; **(C)**The anti-Fn-Dps SIgA titre was detected by ELISA at the indicated times. Data are expressed as mean ± SD.
