# Supplementary material for: Fn-Dps, a novel virulence factor of Fusobacterium nucleatum, disrupts erythrocytes and promotes metastasis in colorectal cancer
Source: PLoS Pathog. 2023 Jan 24;19(1):e1011096. doi: 10.1371/journal.ppat.1011096 (PMC9873182; doi:10.1371/journal.ppat.1011096)
Supplement: S16 Fig — (A) Histopathological examination of the lung tissue sections. (B) Immunohistochemical staining of CCL2, CCL7, E-cadherin and Vimentin expression in paraffin-embedded lung tissues. (C) Histopathological examination of the liver tissue sections. (D) Immunohistochemical staining of CCL2, CCL7, E-cadherin and Vimentin expression in paraffin-embedded liver tissues. Scale bar = 200 μm. (PDF) [file ppat.1011096.s016.pdf]

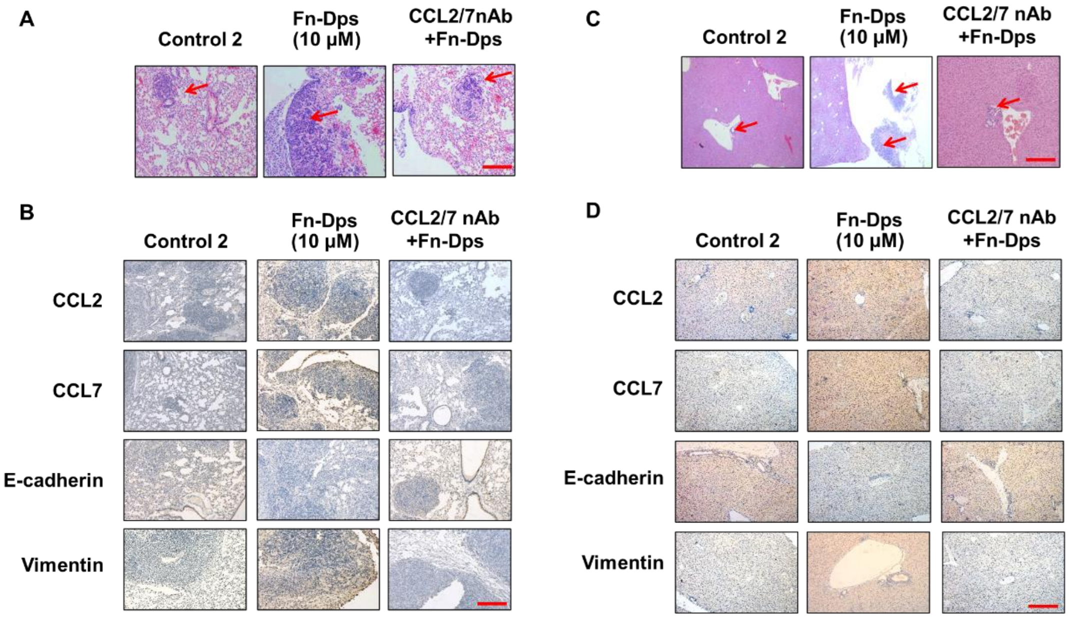

S16 Fig. Fn-Dps promotes the migration of CRC cells *in vivo*. (A) Histopathological examination of the lung tissue sections. **(B)** Immunohistochemical staining of CCL2, CCL7, E-cadherin and Vimentin expression in paraffin-embedded lung tissues**.** **(C)** Histopathological examination of the liver tissue sections**.** **(D)** Immunohistochemical staining of CCL2, CCL7, E-cadherin and Vimentin expression in paraffin-embedded liver tissues. Scale bar = 200 μm.
