# Supplementary material for: Fn-Dps, a novel virulence factor of Fusobacterium nucleatum, disrupts erythrocytes and promotes metastasis in colorectal cancer
Source: PLoS Pathog. 2023 Jan 24;19(1):e1011096. doi: 10.1371/journal.ppat.1011096 (PMC9873182; doi:10.1371/journal.ppat.1011096)
Supplement: S1 Table — (PDF) [file ppat.1011096.s019.pdf]

S1 Table. Sequence of primer.

Gene	Primer 5' - 3'
Fn-Dps	F1: CCATGGGGAAAAACAAAGAAAATT<br>F2: CTCGAGTTTTAGCCATAGCTGAGAT
CCL7	F: CAGAAGGATCACCAGTAGTCGG<br>R: ATAGCCTCCTCGACCCACTTCT
CCL2	F: GCTACAAGAGGATCACCAGCAG<br>R: GTCTGGACCCATTCCTTCTTGG
STFA3	F: CTCTGCTTGAAGAGCAAACCAATG<br>R: TCATGTGAAGGAAACAACCATTCC
CSF3	F: ATCCCGAAGGCTTCCCTGAGTG<br>R: AGGAGACCTTGGTAGAGGCAGA
GAPDH	F: CATCACTGCCACCCAGAAGACTG<br>R: ATGCCAGTGAGCTTCCCGTTCAG
