# Supplementary material for: Fn-Dps, a novel virulence factor of Fusobacterium nucleatum, disrupts erythrocytes and promotes metastasis in colorectal cancer
Source: PLoS Pathog. 2023 Jan 24;19(1):e1011096. doi: 10.1371/journal.ppat.1011096 (PMC9873182; doi:10.1371/journal.ppat.1011096)
Supplement: S2 Table — (PDF) [file ppat.1011096.s020.pdf]

S2 Table. Mass spectrometry (MS)-identified soluble protein of the culture supernatant of Fn under starved conditions.

UniProt Accessions	Description	PSMs
**Q8REM0**	**Neutrophil-activating protein A**	**17**
Q8RI44	Hypothetical protein FN1792	13
Q8RGK1	Hemolysin activator protein	11
Q8RGL0	DNA polymerase III alpha subunit	9
Q8RFC7	Electron transfer flavoprotein beta-subunit	8
Q8R5X7	Full=60 kDa chaperonin;	7
Q8R603	Elongation factor Tu	7
Q8RG20	Hemin receptor	7
Q8RGS8	Glutaconyl-CoA decarboxylase A subunit	6
Q8RI47	Tetratricopeptide repeat family protein	6
Q8RE59	UTP--glucose-1-phosphate uridylyltransferase	5
Q8REJ3	Dipeptide transport ATP-binding protein dppF	5
Q8REY1	Hypothetical protein FN0953	5
Q8R643	Pyruvate-flavodoxin oxidoreductase	4
Q8RG24	Acetyl-CoA acetyltransferase	4
Q8RG30	NAD-specific glutamate dehydrogenase	4
Q8RHI5	RecName: Full=50S ribosomal protein L7/L12	3
Q8RII1	Transcription antitermination protein	3
