# Supplementary material for: Fn-Dps, a novel virulence factor of Fusobacterium nucleatum, disrupts erythrocytes and promotes metastasis in colorectal cancer
Source: PLoS Pathog. 2023 Jan 24;19(1):e1011096. doi: 10.1371/journal.ppat.1011096 (PMC9873182; doi:10.1371/journal.ppat.1011096)
Supplement: S3 Table — (PDF) [file ppat.1011096.s021.pdf]

S3 Table. Mass spectrometry (MS)-identified soluble protein of the culture supernatant of Fn under BHI.

UniProt Accessions	Description	PSMs
Q8RG30	Glutamate dehydrogenase	23
Q8R643	Pyruvate-flavodoxin oxidoreductase	20
Q8RES5	3-hydroxybutyryl-CoA dehydrogenase	18
Q8RFC6	Electron transfer flavoprotein alpha-subunit	18
Q8R6D3	Alkyl hydroperoxide reductase C22 protein	16
Q8RHN0	Thioredoxin reductase	16
Q8REE2	D-galactose-binding protein	15
Q8RI44	Uncharacterized protein	13
Q8RDT9	Threonine dehydratase	12
Q8RHT8	Probable cytosol aminopeptidase	12
Q8RHY1	Major outer membrane protein	11
Q8RE11	Acetoacetate metabolism regulatory protein atoC	11
Q8RHF4	Formate--tetrahydrofolate ligase	11
Q8RIB8	N-acetylneuraminate synthase	11
Q8RDW4	Citrate lyase alpha chain	10
Q8RIJ6	Ion-translocating oxidoreductase complex subunit C	9
Q8R6H3	Probable electron transfer flavoprotein-quinone oxidoreductase ydiS	8
**Q8REM0**	**Neutrophil-activating protein A**	**8**
Q8RFC1	Urocanate hydratase	8
Q8R5Y8	Biotin carboxyl carrier protein of glutaconyl-COA decarboxylase	7
Q8RFL4	Transcriptional regulator, MarR family	7
Q8RGB0	Dipeptide-binding protein	7
Q8RG09	Flavodoxin	7
Q8RFG1	Imidazolonepropionase	7
Q8RET7	Outer membrane protein P1	7
Q8RI54	Pyruvate kinase	7
Q8RDY4	Dipeptide-binding protein	6
Q8RED6	Phosphate acetyltransferase	5
Q8RIN1	Iron-sulfur cluster-binding protein	5
Q8RGH3	Fructose-bisphosphate aldolase class 1	4
