# Supplementary material for: Fn-Dps, a novel virulence factor of Fusobacterium nucleatum, disrupts erythrocytes and promotes metastasis in colorectal cancer
Source: PLoS Pathog. 2023 Jan 24;19(1):e1011096. doi: 10.1371/journal.ppat.1011096 (PMC9873182; doi:10.1371/journal.ppat.1011096)
Supplement: S4 Table — (PDF) [file ppat.1011096.s022.pdf]

S4 Table. Homology analysis of Fn-Dps with *Fusobacterium*.

Description	Accession	Query cover	Identity
F. hwasookii	EJU07968	100%	93%
F. canifelinum	RRD28394.1	100%	93%
F. periodonticum	AVQ24598.1	100%	90%
F. massiliense,	WP_074016696.1	100%	81%
F. subsp animalis	EGQ79128.1	100%	80%
F. russii	WP_022819735.1	100%	69%
F. gonidiaformans,	WP_010680554.1	100%	60%
F. equinum	WP_060793625.1	100%	60%
F. necrophorum	KDE72938.1	100%	58%
