# Supplementary material for: Fn-Dps, a novel virulence factor of Fusobacterium nucleatum, disrupts erythrocytes and promotes metastasis in colorectal cancer
Source: PLoS Pathog. 2023 Jan 24;19(1):e1011096. doi: 10.1371/journal.ppat.1011096 (PMC9873182; doi:10.1371/journal.ppat.1011096)
Supplement: S5 Table — (PDF) [file ppat.1011096.s023.pdf]

S5 Table. Homology analysis of Fn-Dps with other bacteria.

	Description	Accession	Query cover	Identity
obligate anaerobe	*Anaeroglobus geminatus*	WP_040347574.1	97%	52%
	Veillonella parvula	EQC67777	100%	50%
	Colibacter massiliensis	WP_075571901.1	97%	50%
	Halanaerobium sp.MSAO_Bac5	RQD68915.1	98%	52%
	Tindallia sp. MSAO_Bac2	RQD70652.1	98%	52%
	Natronincola ferrireducens	SDL34861.1	98%	51%
	Clostridium isatidis	ASW41992.1	97%	51%
	Alkaliphilus metalliredigens	ABR49594.1	98%	49%
	Tissierella bacterium	NLI65402.1	100%	49%
	Tepidimicrobium xylanilyticum	SDX16139.1	100%	49%
	Anaerococcus hydroalis	KWZ83051.1	97%	48%
	Bacteroides fragilis	RDT77295.1	94%	32%
	Bifidobacterium longum	PWH09146.1	81%	28%
non-obligated anaerobe	*Campylobacter jejuni*	RTK17074.1	86%	38%
	Streptococcus pneumoniae	CJQ05024.1	95%	32%
	Helicobacter pylori	**ADB92490.1**	**97%**	**30%**
	Listeria monocytogenes	PSO41857.1	95%	29%
	Haemophilus influenzae	WP_021035272.1	95%	29%
	Staphylococcus aureus	KZX24682.1	86%	28%
	Salmonella enterica	EDZ5010003.1	86%	28%
	Yersinia pestis	KYP01569.1	86%	28%
	Pseudomonas aeruginosa	AVZ36133.1	95%	27%
	Escherichia coli	ARM39582.1	86%	27%
	Enterococcus faecalis	RTK90555.1	97%	23%
	Lactobacillus brevis	WP_060416942.1	97%	20%
