# Supplementary material for: Fn-Dps, a novel virulence factor of Fusobacterium nucleatum, disrupts erythrocytes and promotes metastasis in colorectal cancer
Source: PLoS Pathog. 2023 Jan 24;19(1):e1011096. doi: 10.1371/journal.ppat.1011096 (PMC9873182; doi:10.1371/journal.ppat.1011096)
Supplement: S6 Table — (PDF) [file ppat.1011096.s024.pdf]

S6 Table. Prediction of antigenic determinants for the Fn-Dps protein among Fn and other related bacteria.

n	*Fusobacterium nucleatum*	*Helicobacter pylori*	*Helicobacter cinaedi*	*Borrelia burgdorferi*	*Mycoplasma pulmonis*
1	NKYLSNLGILITK	FEILKHLQADAIVLSMKVHNF	VVELLKQIQADASVFYVKVHNF	LDAIQLKLQELLASLHIFYSNL	DKLRILQASLTIFNQKLQAYHWNLYGSHFFQWH
2	LHWNVVGARFKAIHEYTESLYDYYF	AERIVQLGHHPLVTLSEALKLTR	IYEQFADVFD	TNFFVIH	QLEKLIKQVR
3	KFDEVAE	ILEDYKHLE	VAERVLQLGEMPYVTLADM	KTQKLYEYIEKIIDIVA	FSISSLEKALEISL
4	EFPLVKVADYLKHATVKE	DKVTVTYADDQLAKLQKS	KTSFCSKEIAQAVLADYEYFLKLFTELSAQ	PSMESIVCSLTEILK	EDVAFDIKHIAKNVVNDIEILLKHIELVSW
5	TIPEVVTS		GDKVSAAYAD	LWMHKALLENCDCFC	TEQPLIDEIILGL
6	IEYFVKQLWFI		KVGELQKAIWML		
**n**	***Treponema pedis str.***	***Vibrio nigripulchritudo***	***Veillonella parvula***	***Lactobacillus ruminis***	***Fusobacterium necrophorum***
1	FNQYLADLAVITFKLHNLHWN	TLIGLDQ	VQQVNQYLADLSVWNVKLHNLHFNVTG	SKSGCAASKVEQ	VELLNKYLSNLAVLIVKLHNLHWNVVGQ
2	EFMAIHQFTESVYD	DLAKALNTLLANYQVLY	QFKSIHEYLES	KLGKCST	YDTYFGYYDDVAE
3	VMPDCKLSDYLAN	GHAFFEL	EYFDAVAEH	FYSFAFAKAVWS	QRPLVRM
4	EIKEVDA	IYTDLQTKVDE	GQFPLVNS		DYLAVTSI
5	TAKEVLQIVKNDLTVLR	AERILTLGHTPLHSFTEYLEVST	PQAKVIDILLKD		NFSPCEVLSIVKA
6	SAATLFDEHVQYYNKQLWF	MKGLVKGFSQLISQQRDILGL	KDQAVAIR		
7		QEKLVWM	DHVAYYVKQIW		
