# Supplementary material for: Fn-Dps, a novel virulence factor of Fusobacterium nucleatum, disrupts erythrocytes and promotes metastasis in colorectal cancer
Source: PLoS Pathog. 2023 Jan 24;19(1):e1011096. doi: 10.1371/journal.ppat.1011096 (PMC9873182; doi:10.1371/journal.ppat.1011096)
Supplement: S7 Table — (PDF) [file ppat.1011096.s025.pdf]

S7 Table. Relationship between the OD value of IgG/IgA antibodies against Fn-Dps and the clinicopathological variables in 123 patients with CRC.

**Characteristics**		**cases (n)**	**IgG (Means±SD)**	***P***	**IgA (Means±SD)**	***P***
**Gender**						
	Male	64	0.58±0.32	0.27	0.52±0.38	0.38
	Female	59	0.64±0.32		0.58±0.39	
**Age(y)**						
	<60	71	0.59±0.31	0.33	0.53±0.39	0.45
	≥60	52	0.64±0.34		0.58±0.38	
**Tumor volume (cm^3)**						
	<5	71	0.60±0.33	0.16	0.50±0.37	0.09
	≥5	52	0.63±0.30		0.62±0.40	
**Grade**						
	G1+G2	111	0.65±0.36	0.33	0.59±0.41	0.05
	G3+G4	12	0.51±0.27		0.34±0.28	
**Stage**						
	I	27	0.47±0.23	**0.02**	0.38±0.24	**0.01**
	II+III+IV	96	0.65±0.33		0.59±0.39	
**T status**						
	T1+ T2	30	0.53±0.36	0.09	0.62±0.39	0.30
	T3+T4	93	0.65±0.33		0.53±0.38	
**N status**						
	No	55	0.56±0.31	0.10	0.50±0.39	0.20
	Yes	68	0.66±0.33		0.59±0.38	
**Metastasis**						
	No	99	0.61±0.33	0.78	0.55±0.40	0.86
	Yes	24	0.63±0.30		0.56±0.35	
**CEA (μg/ml)**						
	<5	69	0.58±0.34	0.19	0.54±0.40	0.84
	≥5	54	0.65±0.33		0.56±0.37	
**CA19-9 (U/ml)**						
	<35	98	0.60±0.32	0.30	0.54±0.40	0.45
	≥35	25	0.57±0.31		0.51±0.31	
